# *In silico* target identification and pharmacokinetic profiling of 2-aryl-quinoline-4-carboxylic acid derivatives as potential antileishmanial agents

**DOI:** 10.3389/fphar.2025.1621059

**Published:** 2025-07-21

**Authors:** Marília Cecília da Silva, Jéssika de Oliveira Viana, Tayná Rodrigues Olegário, Jayne Maria Sabino, Euzébio Guimarães Barbosa, Elton José Ferreira Chaves, Gerd Bruno Rocha, Claudio Gabriel Lima-Junior, Karen Cacilda Weber

**Affiliations:** ^1^ Computational Quantum Chemistry Laboratory, Department of Chemistry, Federal University of Paraíba, João Pessoa, Brazil; ^2^ Medicinal Organic Synthesis Laboratory of Paraíba (LASOM-PB), Department of Chemistry, Federal University of Paraíba, João Pessoa, Brazil; ^3^ Computational Pharmaceutical Chemistry Laboratory, Faculty of Pharmacy, Federal University of Rio Grande do Norte, Natal, Brazil

**Keywords:** inverse virtual screening, molecular docking, molecular dynamics, ADMET properties, similarity search

## Abstract

**Introduction:**

Leishmaniasis remains a major neglected tropical disease, and new therapeutic strategies are urgently needed. This study aimed to identify the molecular target of 2-aryl-quinoline-4-carboxylic acid derivatives and assess their pharmacokinetic profiles.

**Methods:**

An integrated *in silico* workflow was employed, including inverse virtual screening (IVS), molecular docking, molecular dynamics (MD) simulations, and ligand-based similarity searches in public chemical databases. Pharmacokinetic and toxicity predictions were also performed.

**Results:**

IVS highlighted *Leishmania major* N-myristoyltransferase (*Lm*NMT) as the most frequent high-affinity target. Docking and MD simulations demonstrated stable binding of selected compounds, with compound **2d** showing the highest docking scores and compound **1g** displaying enhanced affinity after conformational relaxation of the enzyme. Ligand-based similarity search confirmed the superior predicted binding affinity of the studied compounds compared to known molecules. Most derivatives exhibited favorable predicted pharmacokinetic properties and comparable or improved profiles relative to DDD85646.

**Discussion:**

These results support the potential of the 2-aryl-quinoline-4-carboxylic acid scaffold as a basis for the development of novel *Lm*NMT inhibitors with promising pharmacokinetic properties, paving the way for further experimental validation.

## 1 Introduction

Leishmaniasis, a neglected tropical disease, is caused by several species of *Leishmania* and has three different clinical forms: cutaneous, mucocutaneous and visceral (Hussain et al., 2014). The visceral form, also known as kala-azar, is the most serious, affecting vital organs such as the liver, spleen and bone marrow, and is frequently associated with the *Leishmania donovani* and *Leishmania infantum* species (Cardoso et al., 2015). The current treatment is based on pentavalent antimonials, Amphotericin B and miltefosine, however, these drugs face significant limitations, including high toxicity, serious side effects and increasing parasite resistance (Abongomera et al., 2018). These challenges highlight the need for new, more efficient and safer therapeutic agents for the control of leishmaniasis.

Quinoline derivatives have emerged as promising candidates with leishmanicidal potential (Chanquia et al., 2019; Ibrahim et al., 2023). Quinolines, recognized for their structural diversity and broad spectrum of biological activities, have been widely investigated in organic synthesis (Zarghi et al., 2009). These compounds have significant pharmacological relevance, with antifungal, antibacterial and anticancer properties (Fournet et al., 1996), among other bioactivities. Quinolines also demonstrate antileishmanial activity, as evidenced by sitamaquine, a trisubstituted quinoline which has reached phase 2 clinical trials for the treatment of visceral leishmaniasis (Gopinath et al., 2013; Nakayama et al., 2007). A series of 15 quinoline-4-carboxylic acid analogues were investigated by Abdelwahid et al. (2019) against the *L. donovani* promastigote (clinical isolate) at different concentration levels with two drugs used in the treatment of leishmaniasis (sodium Stibogluconate and Amphotericin B) as positive controls (Abdelwahid et al., 2019).

In drug design, computational methods have become indispensable tools, reducing costs and optimizing the drug discovery process by identifying promising biological targets and assisting in the design of new bioactive compounds. These methods have consolidated their relevance in theoretical and medicinal chemistry. It is possible to map regions of molecular interaction that are crucial to understanding the ligand’s mode of action based on information about the 3D structure of the target protein (De Vivo et al., 2016). In this scenario, *in silico* target fishing techniques, such as Inverse Virtual Screening (IVS), have gained prominence for their versatility and are widely used in the discovery of new drugs. This approach uses a molecule as bait to identify possible targets and associated biological activities, and can be conducted based on the structure of the ligand or receptor, expanding the possibilities in rational drug design (Galati et al., 2021).

*In silico* identification of biological targets has proven to be a promising alternative to drug design. Most traditional approaches are based on trial and error or costly and sophisticated experimental methods, limiting their applicability on a large scale. In contrast, *in silico* techniques make it possible to identify molecular targets with greater affinity and understand ligand-receptor interactions, optimizing the design of new derivatives with improved therapeutic properties (Galati et al., 2021). Numerous molecular targets have been identified against *Leishmania* species, with their structures determined by X-ray crystallography or cryo-EM, enabling the use of computational methods (Jones et al., 2018). However, just a few have employed computational target fishing strategies to identify the most promising targets for small molecules with reported antileishmanial activity (Alamzeb et al., 2021; Almeida et al., 2021; Bayraktar et al., 2024).

Integrating several *in silico* approaches, this study aimed to identify a putative target for a series of 2-aryl-quinoline-4-carboxylic acid derivatives. A small dataset of reference compounds, including a newly synthesized compound inspired by the findings of Muscia et al. (2008) and Abdelwahid et al. (2019), was used as controls to characterize the interaction profile with the identified target, supporting the selection of N-myristoyltransferase (NMT) as the most promising one. Additionally, a ligand-based similarity search in publicly available databases confirmed that the studied series exhibits higher predicted affinity for NMT compared to known compounds. Altogether, our findings provide a structure-based platform for the development of novel antileishmanial agents based on the quinoline scaffold.

## 2 Materials and methods

### 2.1 Dataset

Based on the reports from Muscia et al. (2008), Abdelwahid et al. (2019), and Olegário et al. (2025), a total of 15 compounds were selected as a dataset (Supplementary Table S1). Additionally, a new molecule (compound **1g**) was proposed based on the common scaffold of this compound series, bringing the total to 16 compounds. The three-dimensional structures of the compounds were created using the MarvinSketch (MarvinSketch, 2025) and OpenBabel software (O’Boyle et al., 2011), considering the physiological pH of 7.4 for protonation, as it is commonly used in *silico* studies to approximate physiological conditions. MOPAC software was used to optimize the ground state geometries using the PM6 semi-empirical method (Stewart Computational Chemistry (Stewart, 2016), http://www.OpenMOPAC.net).

### 2.2 General procedure for the synthesis of the query molecule 2-([1,1′-biphenyl]-4-yl)-6,8-dichloroquinoline-4-carboxylic acid (1g)

Based on the findings of Muscia et al. (2008) and Abdelwahid et al. (2019), we designed a new molecule bearing a biphenyl moiety and two chlorine atoms in the 6 and 8 positions of the quinoline portion (see Supplementary Table S1), to be used as a query molecule for the inverse virtual screening tests. These modifications in the scaffold of the series under study were devised to explore strategic positions in the binding site indicated by Abdelwahid et al. (2019). In a round-bottomed flask, 0.5 mmol of 5,7-dichloroisatin was solubilized in 2.5 mL of 30% KOH(aq). After stirring the resulting solution for 5 min at room temperature, 1.0 mmol of 4-acetylbiphenyl was added. The reaction mixture was refluxed until completion confirmed by TLC and subsequently acidified with 10% HCl(aq) to pH 5 after cooling. The precipitate was then filtered under vacuum and washed with ice water. The crude product was then purified by recrystallization from ethanol.

2-([1,1′-biphenyl]-4-yl)-6,8-dichloroquinoline-4-carboxylic acid (1g): Yellow powder. Yield 97%. 1H NMR (400 MHz, DMSO-d6) δ in ppm: 8.71 (t, J = 2.0 Hz, 1H); 8.63 (t, J = 1.7 Hz, 1H); 8.41 (m, 2H); 8.12 (m, 1H); 7.87 (m, 2H); 7.77 (m, 2H); 7.52 (m, 2H); 7.43 (m, 1H). 13C NMR (100 MHz, DMSO-d6) δ in ppm: 167.3; 156.4; 143.5; 142.5; 139.6; 137.8; 136.5; 135.0; 132.0; 130.7; 129.5; 129.5; 129.3; 128.5; 128.4; 127.7; 127.4; 127.3; 127.2; 125.7; 124.3; 121.5.

### 2.3 Inverse virtual screening

A representative compound of the series (compound **1g**, Supplementary Table S1) was selected to perform the Inverse Virtual Screening (IVS) protocol proposed by de Oliveira Viana and coworkers (2023). In this technique, a non-redundant library of approximately 23,000 protein structures was systematically retrieved from the RCSB Protein Data Bank (Berman et al., 2002) using predefined criteria designed to represent a diverse set of biological targets. The downloaded data were manually inspected to ensure the absence of duplicates, with docking simulations being performed on all crystallized ligand-binding sites of the selected proteins, as defined in the PDB structures, using Autodock Vina (Trott and Olson, 2010) with *ad hoc* scripts for workflow automation. The resulting data were sorted according to docking scores and analyzed by human inspection to determine the priority order of potential biological targets.

### 2.4 Protein preparation

Once the IVS approach has identified N-myristoyltransferase (NMT) as the highest scored enzyme, their *Leishmania major* and *L. donovani* structures were retrieved from the RCSB Protein Data Bank (PDB). The loops and missing residues of the enzymes - PDB codes 2WSA (Frearson et al., 2010) and 2WUU (Brannigan et al., 2010), respectively - were reconstructed using the Modeller 11.2 software (Šali and Blundell, 1993). Protonation states of charged residues were subsequently adjusted to reflect a physiological pH of 7.4 using the H++ server (Gordon et al., 2005).

### 2.5 Pharmacophoric modeling

To evaluate the reproduction of crystallographic poses and the discrimination between active and inactive compounds, the GOLD software was used to define pharmacophoric models, considering four scoring functions. In view of the high sequence identity (97.8%) between the N-myristoyltransferase enzymes from *L. major* (*Lm*NMT) and *L. donovani* (*Ld*NMT) (Brannigan et al., 2014), four pharmacophoric models were developed for *Lm*NMT. These models were generated from the crystallographic structures of protein-ligand complexes available on the PDB, with the corresponding codes: 2WSA (Frearson et al., 2010), 4CGN (Brannigan et al., 2014), 5A28 (Rackham et al., 2015) and 5G21 (Goncalves et al., 2017). The NMT enzyme PDB 2WSA is co-crystallized with a pyrazole sulfonamide inhibitor (DDD85646), a potent N-myristoyltransferase inhibitor against *Trypanosoma brucei* (Brand et al., 2012). NMT enzymes PDB 4CGN and 5A28 include experimental Ki data for their respective crystallographic ligands (Brannigan et al., 2014; Rackham et al., 2015), while PDB 5G21 features a quinoline inhibitor with demonstrated activity against *Plasmodium vivax* and *Plasmodium falciparum* NMT (Goncalves et al., 2017). The models were built based on the main interactions between the ligands and the residues from the active site, identified from the 2D diagrams generated in BIOVIA Discovery Studio v2021 (BIOVIA, 2021). More stringent restrictions were applied to regions of π-π interactions compared to hydrogen bonding interactions.

### 2.6 ROC curve and dataset preparation

The Receiver Operating Characteristic (ROC) curve was used to validate the pharmacophoric models, comprising a statistical approach that evaluates the sensitivity and specificity of the models in discriminating between active compounds (true positives) and decoys (false positives). In this analysis, the area under curve (AUC) is a performance metric that summarizes the trade-off between sensitivity (true positive rate) and specificity (false positive rate) across all classification thresholds. An AUC of 1.0 indicates perfect discrimination between classes, while an AUC of 0.5 suggests no discriminative power, equivalent to random guessing. This method allowed a detailed analysis of the effectiveness of the GOLD scoring functions (Empereur-Mot et al., 2016). Thus, the validation database was set up by searching for compounds interacting with the target enzyme determined by the IVS (NMT enzyme), for *L. major* organisms, in the ChEMBL database (Gaulton et al., 2012). Only molecules with experimental Ki data were considered, and they were classified as active (pKi >9) or inactive (pKi <8), taking as reference the Ki value of the crystallographic ligand bound to the NMT enzyme - PDB code 5A28 (Rackham et al., 2015). The screening identified 21 compounds (Supplementary Table S2), of which 9 were classified as active (Ki values between 1.3 nM and 8.5 nM) and 12 as inactive (Ki values between 13.9 nM and 2,600.0 nM). Approximately 650 decoys were produced from the active structures, all of which were included in the analysis. Subsequently, the three-dimensional structures of the active and inactive compounds and the decoys were constructed using OpenBabel (O’Boyle et al., 2011), with protonation states adjusted to a pH of 7.4.

### 2.7 Ligand-receptor docking

To perform the ligand-receptor docking studies, the binding sites were defined based on the residues at 10 Å from the ligand bound to the enzyme structure and on the coordinates of the crystallographic ligand center of mass, for each *Lm*NMT enzyme. All water molecules were removed from the binding sites. The standard docking protocol was applied (automatic settings, 10 rounds of the genetic algorithm), along with optimized settings determined for maximum efficiency (200%), and exploiting the flexibility of the ligand in the receptor. A consensus analysis was conducted to identify the 2-aryl-quinoline-4-carboxylic acid derivatives with the highest affinities for *Lm*NMT (PDB 2WSA), using the GOLD software with four scoring functions. The results were normalized using the rank-by-number method (Blanes-Mira et al., 2022), combining multiple scoring functions to calculate an average of the values obtained.

### 2.8 MD simulations

MD simulations of the ligand-protein complexes formed by *Lm*NMT (PDB code 2WSA) and ligands **1g** (the novel synthesized compound), **2d** (the highest affinity compound) and **DDD85646** (co-crystallized ligand, used as reference) were carried out using Gromacs v. 2021.2 (Abraham et al., 2015). The best pose predicted by the molecular docking was employed as a starting structure for the simulations. Ligands and cofactors were parameterized according to the GAFF2 force field (Wang et al., 2004), and their partial atomic charges were calculated with the AM1-BCC method using the Antechamber program (Wang et al., 2006). The AMBER99SB-ILDN force field (Lindorff-Larsen et al., 2010) was employed for the protein, along with the TIP3P water model. Ligand-receptor complexes were embedded in a 15 Å cubic simulation box. The net charge of the system was neutralized by adding sodium and chloride ions (0.15 M). Energy minimization of the systems was conducted using the steepest descent algorithm, followed by gradual heating under constant volume and temperature (NVT) conditions. System temperature was increased incrementally from 300 K to 315 K over a 500 ps period, with adjustments made in 5 K intervals to ensure a smooth equilibration process. The thermalization (NVT) and pressurization (NPT) steps at a constant temperature of 300 K were conducted right after heating the systems, for 200 and 350 ps, respectively, with the temperature chosen according to the *in vitro* study by Khalil et al. (2019). The LINCS algorithm was employed to treat intramolecular bonding constraints in all steps, and the particle mesh Ewald (PME) method was used for long-range Coulombic interactions. Temperature and pressure were controlled by a Nosé-Hoover thermostat and a Parrinello-Rahman barostat. After the equilibration steps, 200 ns of production simulations were run for each system, in triplicates generated by setting different random initial velocities. The analysis tools of CPPTRAJ (Roe and Cheatham, 2013) were used to evaluate the structural and dynamic properties along the trajectories, such as RMSD and RMSF. Trajectory clusterization was conducted using the *gromos* clustering algorithm, with a cutoff radius of 0.2 nm, to evaluate the most representative conformations of the MD simulation trajectories. The most representative structure of the most populated cluster proceeded to perform molecular docking using GOLD, after a structural minimization with the AM1 semiempirical method by using UCSF Chimera (Pettersen et al., 2004).

### 2.9 ADMET predictions

The smile codes of each compound were used as input in SwissADME (Daina et al., 2017) and pkCSM (Pires et al., 2015) to calculate pharmacokinetic, pharmacochemical, and drug likeness properties of these compounds. The following parameters were analyzed through the SwissADME server: Consensus LogP, solubility (Silicos-IT), gastrointestinal absorption (GA), blood-brain barrier (BBB) permeability, P-glycoprotein (P-gp) substrate, and inhibition of the cytochrome P450 isoform (CYP3A4). Using the pkCSM platform, pharmacokinetic properties of small molecules were predicted based on graph-based signatures. The following toxicity parameters were analyzed: hepatotoxicity and the AMES test.

### 2.10 Ligand-based similarity search

In order to search for compounds structurally related to our series and possible higher affinity to NMT, a ligand-based similarity search using two reference compounds (**1g** and **2d**) was conducted in the following publicly available databases: ChEMBL, ZINC, and PubChem. The ChEMBL database search (Zdrazil et al., 2024) retrieved 39 molecules with ≥60% similarity to high-affinity compounds. The ZINC database search (Irwin and Shoichet, 2005) yielded 9,641,447 molecules, with a subset selected using the tranches system based on criteria such as 3D representation, standard reactivity, neutral charge, availability (in stock), and pH 7.4. Two filtrations were applied using OpenBabel (O’Boyle et al., 2011), substructure-based screening followed by similarity screening based on top-performing quinoline structures, using a Tanimoto coefficient threshold of 0.6 (60% similarity) (Bajusz et al., 2015). The PubChem database search (Kim et al., 2023), retrieved 1,220 molecules using a 2D fingerprint Tanimoto similarity-based search, selecting compounds with ≥90% 2D similarity to previously identified high-affinity compounds. The same ADMET properties calculated for the 2-aryl-quinoline-4-carboxylic acid derivatives dataset were also calculated for all compounds obtained from the screening in order to select only those molecules with an adequate pharmacokinetic profile, using the SwissADME and pkCSM tools. Molecular docking was performed on molecules having a suitable pharmacokinetic profile, using the same protocols adopted for the 2-aryl-quinoline-4-carboxylic acid derivatives series (Supplementary Table S1).

## 3 Results

### 3.1 Synthesis of compound 1g

The novel quinoline-4-carboxylic acid **1g** was obtained in good to excellent 97% yields via a Pfitzinger reaction between 5,7-dichloroisatin and 4-acetylbiphenyl, following the protocol described in the literature (Olegário et al., 2025). This compound was characterized by IR, ^1^H, and ^13^C NMR spectroscopy. The formation of the quinoline ring was confirmed by the presence of the most unshielded singlet about 8.6 ppm in the ^1^H NMR spectra (Supplementary Figure S1a), which refers to the quinoline hydrogen closest to the carbonyl group and by the signals near 143 and 156 ppm in the ^13^C NMR spectra, characteristic of carbons neighboring the quinolinic nitrogen (Supplementary Figure S1b).

### 3.2 NMT as a putative target for quinoline derivatives

Providing additional evidence for the antileishmanial potential of the quinoline derivatives reported previously (Muscia et al. (2008); Abdelwahid et al. (2019), the IVS approach indicated that the N-myristoyltransferase enzyme from *L. major* (*Lm*NMT) appears 24 times, presenting binding energy estimates ranging from −12.3 kcal/mol to −9.3 kcal/mol (Supplementary Table S3).

**FIGURE 1:**
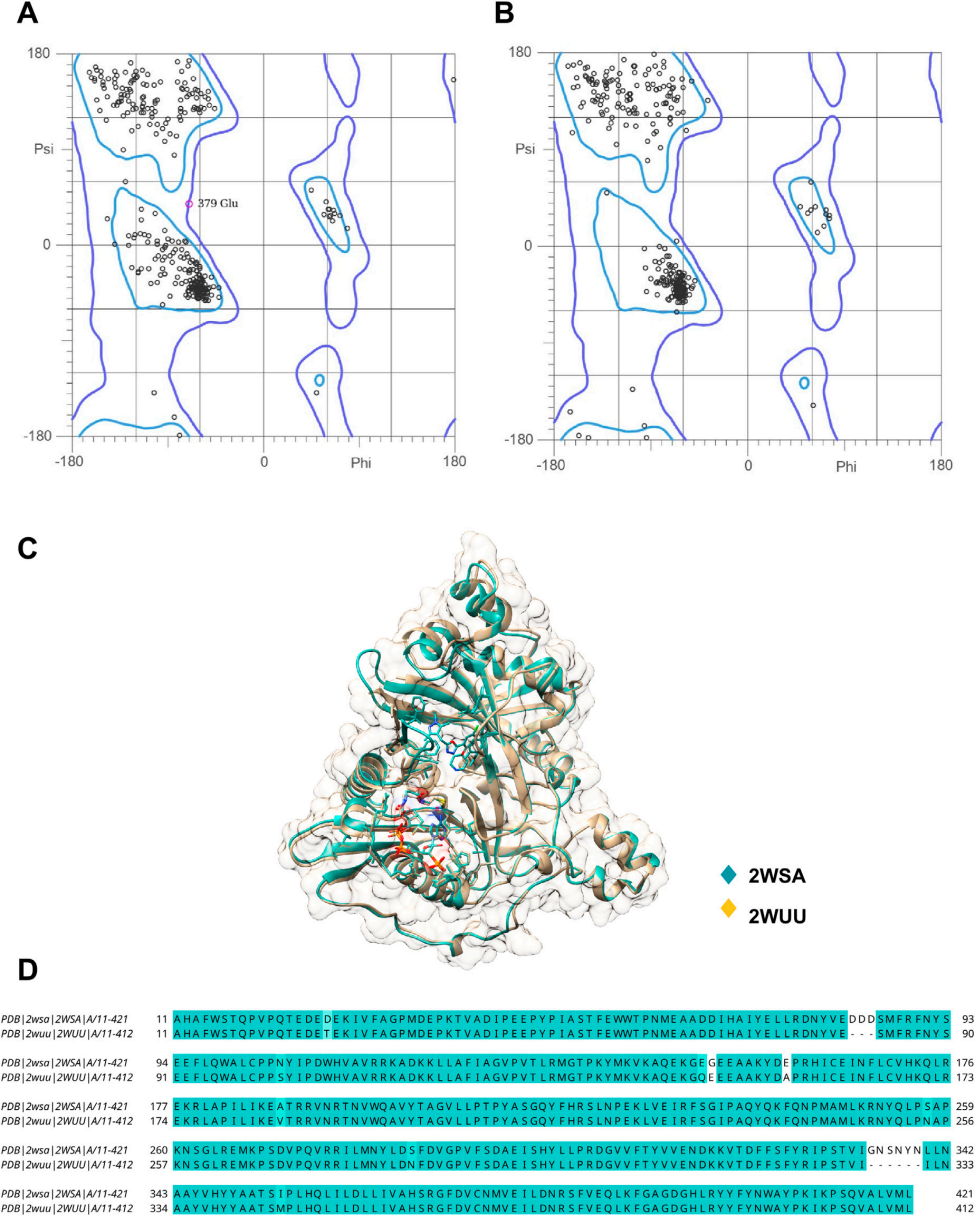
Ramachandran plot of **(A)** *Lm*NMT (PDB 2WSA) and **(B)** *Ld*NMT (PDB 2WUU) showing the distribution of the φ and ψ angles of the amino acid residues. **(C)** 3D structure of the superimposed proteins 2WSA (cyan) and 2WUU (beige), showing the structural similarity between the two proteins. **(D)** Sequence alignment of the proteins 2WSA and 2WUU, highlighting the conserved (light blue) and identical (cyan) residues.

Although some of the molecules of our dataset have biological activity reported for *L. donovani* strains (Abdelwahid et al., 2019), most of the targets identified in our study were related to *L. major* organisms, with N-myristoyltransferase being considered the most promising target for this species due to its lower binding affinity values calculated by IVS. Due to the high identity between the *L. major* and *L. donovani* sequences, which vary between 90% and 98%, the docking studies were conducted based on the *L. major* enzymes, as well as the entire validation protocol of this study, since *L. major* enzymes present experimental Ki data reported in the literature for their crystallographic ligands (Brannigan et al., 2014; Rackham et al., 2015). In fact, *Lm*NMT shares 97% of identity with *Ld*NMT and 100% of identity in the binding site (Figure 1).

### 3.3 Pharmacophoric modelling and ROC curve for evaluating the docking protocol

**FIGURE 2:**
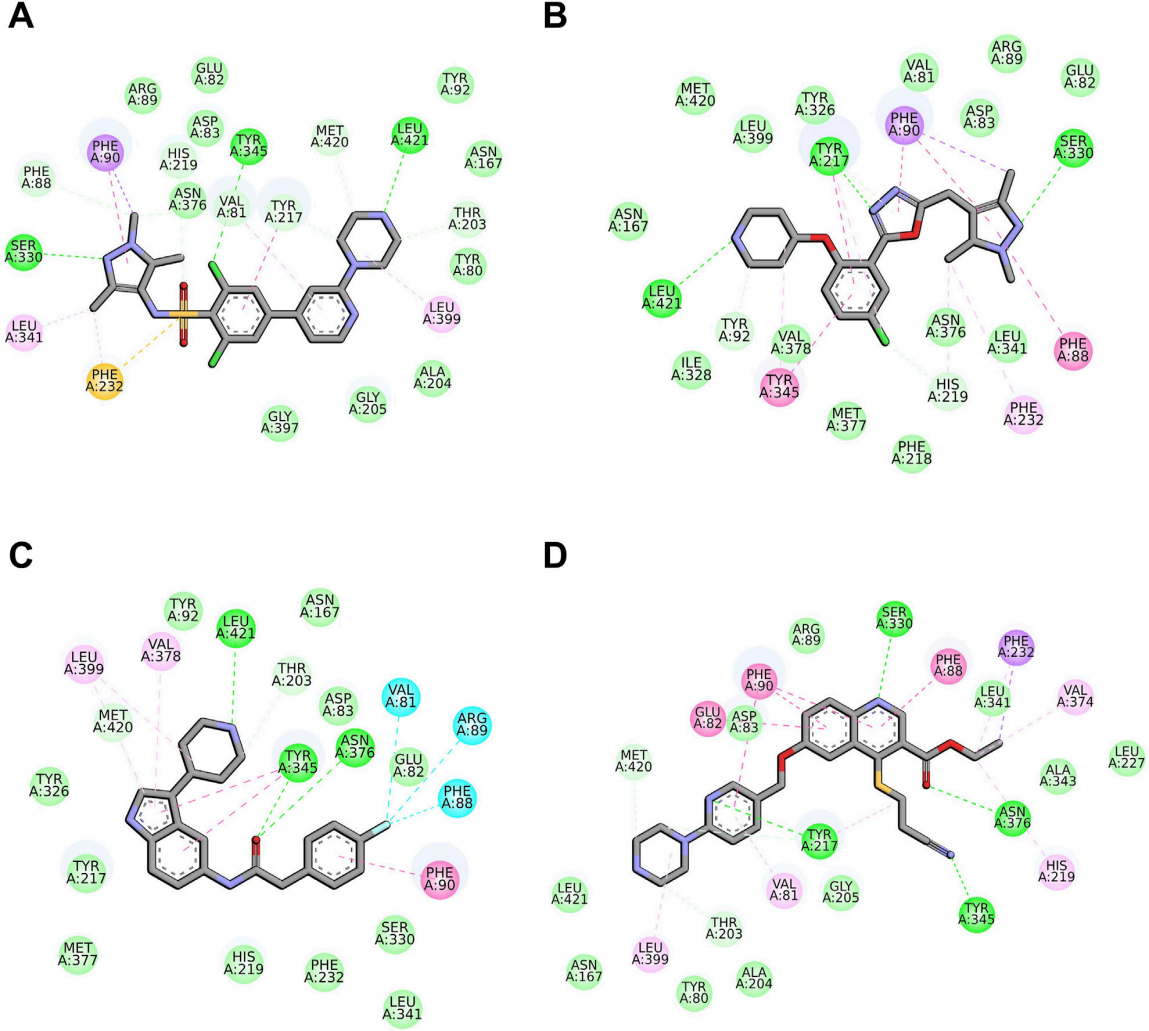
Main interactions between *Lm*NMT binding site residues and crystallographic ligands for each PDB entry: **(A)** 2WSA; **(B)** 5A28; **(C)** 4CGN and **(D)** 5G21. The color of interactions is represented by van der Walls in light green, conventional hydrogen bonds in lime green, π-sigma in purple, π-sulfur in orange, π - π stacked in pink and π-alkyl in light pink.

The docking protocol was evaluated using the ROC curve as a statistical parameter, using four pharmacophoric models developed in GOLD, based on the key interactions between ligands and binding site residues of different PDB entries for the *Lm*NMT enzyme. The main ligand-receptor interactions were examined (Figure 2). It was possible to observe that residues Tyr217, Phe90, Asn376, Tyr345 and Leu421 are involved in different types of interactions with the crystallographic ligands, as presented in Table 1. The residues Tyr217 and Tyr345, highlighted in the literature as crucial for *L. major* (Brannigan et al., 2014), were defined as key constraints for model construction.

**TABLE 1:** Types of interaction between protein residues and crystallographic ligands for each PDB entry (2WSA, 4CGN, 5G21 and 5A28).

Types of interaction^a^				
Residue	2WSA	4CGN	5G21	5A28
Tyr217	Hb	π - π	π - π	-
Phe90	Hm	π - π	Hm	π - π
Phe88	π - π	-	π - π	-
Ser330	Hb	-	Hb	Hb
Asn376	-	Hb	-	Hb
Tyr345	Hb	Hb	π - π	Hm
Val81	Hb	Hb	-	-
Leu421	Hb	Hb	-	Hb
His219	Hb	-	Hb	-

^a^ Hb - Hydrogen bonds, π - π interactions e Hm - multiple interactions (Hb and π - π).

**FIGURE 3:**
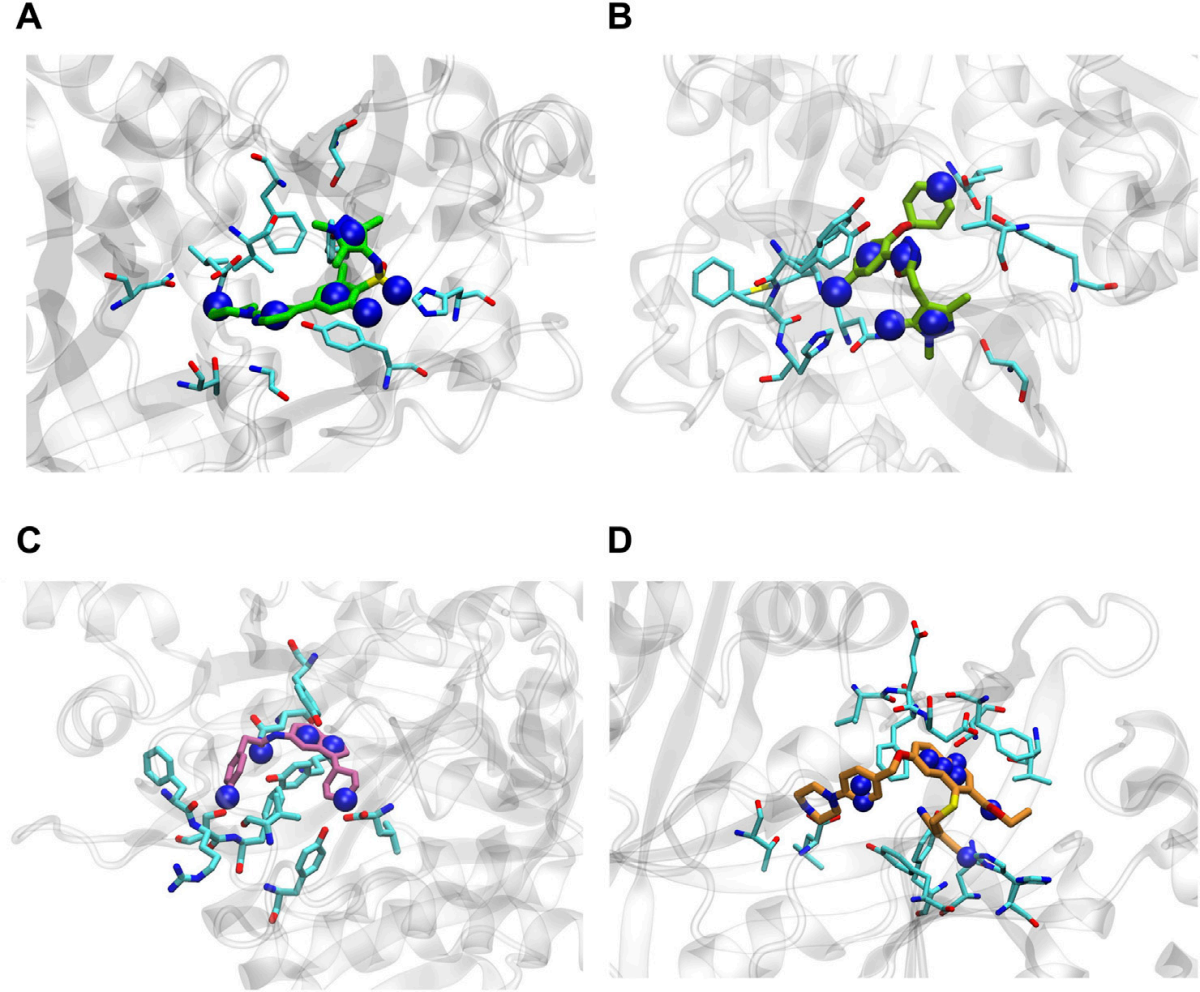
Pharmacophoric spots of each PDB ligand, for the four different PDB entries: **(A)** 2WSA; **(B)** 5A28; **(C)** 4CGN and **(D)** 5G21. The blue balls represent the pharmacophoric spots defined. Carbon atoms of ligands are colored green for A and B, pink for C and orange for D, while carbon atoms for amino acids are colored cyan. Oxygen atoms are represented in red, nitrogen in blue and sulfur in yellow.

The pharmacophoric spots defined based on the interactions observed range from 5 to 8 attributes per model (Figure 3). The model based on PDB 2WSA includes six attributes, highlighting interactions with Tyr217, Leu421, Val81, Tyr345, His219 and Phe90. The PDB 4CGN model features five attributes, focusing on Leu421, Tyr345 (Hm) and Asn376. For PDB 5A28, six attributes were defined, with the main interactions involving Leu421, Tyr345 (Hm), Tyr217 (Hm), Phe90 and His219. The PDB 5G21 model, with eight attributes, includes interactions with Ser330, Phe90, Phe88, Glu82, Tyr217, Tyr345 and Asn376, also incorporating residues related to halogenated substituents present in the quinoline compounds.

**FIGURE 4:**
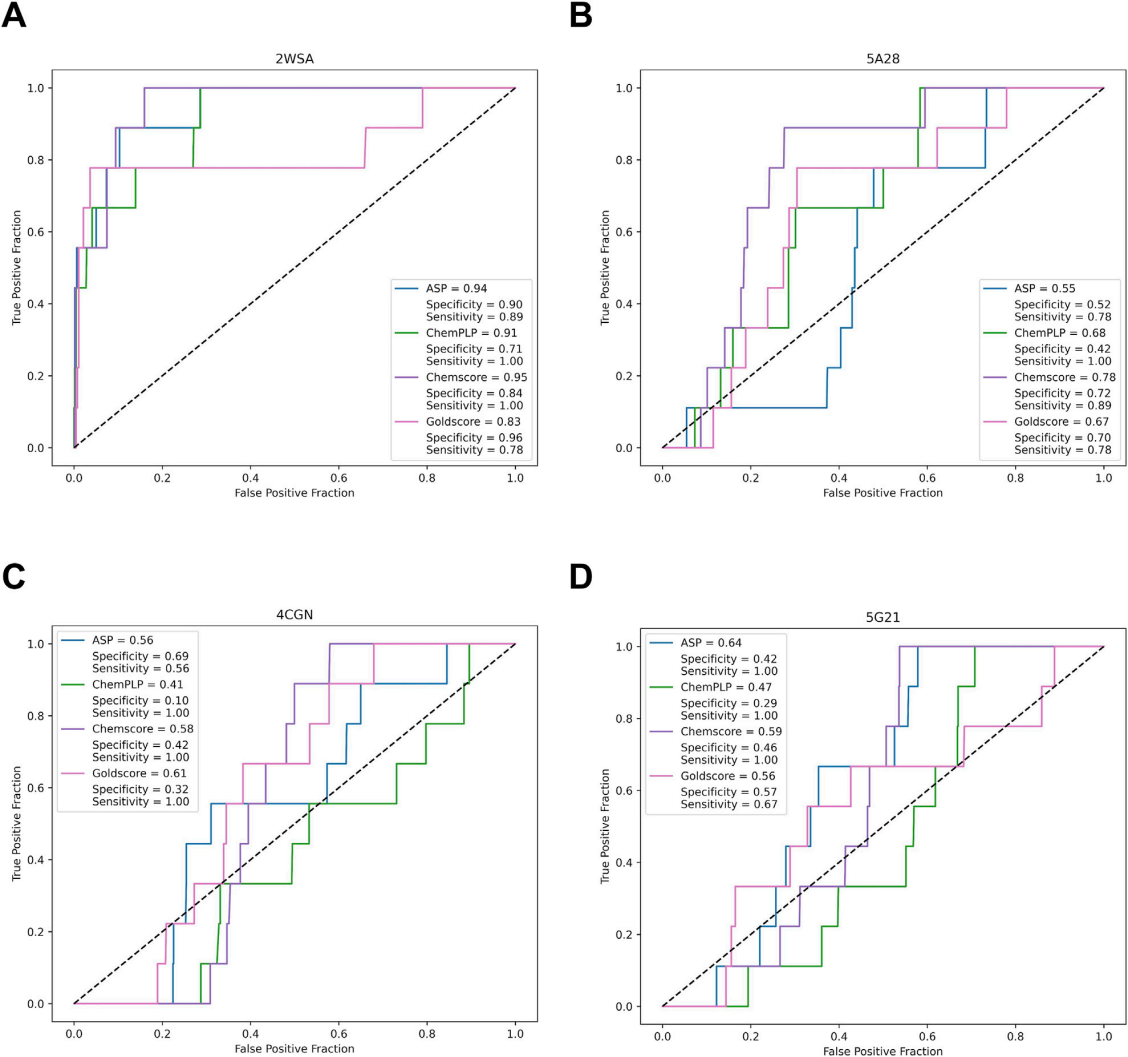
ROC curves and calculation of area under curve (AUC), specificity and sensitivity for the pharmacophoric models produced by each of the GOLD scoring functions (ASP, ChemPLP, Chemcore and Goldscore) for each PDB entry: **(A)** 2WSA; **(B)** 5A28; **(C)** 4CGN and **(D)** 5G21.

The ROC curves generated for each pharmacophoric model and each scoring function to evaluate the AUC of the models are shown in Figure 4. The model associated with the PDB code 2WSA demonstrated sensitivity values ranging from 0.78 to 1.0, with the ChemPLP and Chemscore scoring functions achieving the highest values. The specificity of this model was also consistent, ranging from 0.71 to 0.96, emphasizing its high accuracy in excluding inactive compounds. The models for PDB codes 4CGN and 5G21 showed lower overall values but demonstrated high sensitivity for certain scoring functions. The PDB 5A28 model displayed intermediate sensitivity and specificity, with Chemscore and Goldscore yielding the most consistent results.

Based on the AUC values, the model designed for the PDB code enzyme 2WSA presented the best results, with AUCs higher than 0.9 for most of the scoring functions, except Goldscore. This indicates a higher probability of assigning higher scores to active compounds than to inactive ones, showing a non-random classification. The Chemscore function, which provided the best AUC results, was chosen for subsequent analyses. The pharmacophoric restrictions defined for this model were also applied to represent the binding modes in the molecular docking at the *Ld*NMT. As an additional evaluation of the accuracy of GOLD scoring functions, the best-docked ligand conformation in the *L. major* NMT (PDB 2WSA) was compared to its crystallographic pose using RMSD calculations in PyMol (Schrödinger, LLC) (PyMOL, 2025). Results are shown in Table 2. All scoring functions yielded favorable RMSD results, with values below 1.0 Å.

**TABLE 2:** RMSDs of the docked ligand structures compared to the crystallographic pose of the *Lm*NMT (PDB code 2WSA) ligand.

Scoring function	RMSD (Å)
ASP	0.651
ChemPLP	0.851
Chemscore	0.700
Goldscore	0.664

### 3.4 Molecular docking at LmNMT and LdNMT

Following these outcomes, docking simulations for *Lm*NMT were performed using all GOLD scoring functions using a consensus approach, aiming to identify quinoline compounds with optimal interactions with *Lm*NMT. The results are shown in Table 3, where it is possible to observe that compound **2d** is the best scored ligand for *Lm*NMT.

**TABLE 3:** Consensus analysis of docking scores at *Lm*NMT (PDB code 2WSA) using the rank by number method.

ID	ASP	nASP	PLP	nPLP	CHS	nCHS	GS	nGS	nG
1a	70.09	0.712	97.46	0.771	70.45	0.787	85.38	0.803	0.768
1b	70.61	0.717	96.54	0.764	67.46	0.754	86.96	0.818	0.763
1c	68.39	0.695	95.91	0.759	70.56	0.789	88.58	0.833	0.769
1d	71.68	0.728	95.03	0.752	70.01	0.782	89.02	0.837	0.775
1e	70.72	0.718	75.06	0.594	67.75	0.757	90	0.847	0.729
1f	70.67	0.718	73.52	0.582	70.91	0.793	91.78	0.863	0.739
1g	70.6	0.717	96.97	0.767	72.53	0.811	94.03	0.884	0.795
2a	89.21	0.915	115.84	0.920	86.78	0.932	105.83	0.936	0.926
2b	92.08	0.944	114.16	0.906	89.51	0.961	107.45	0.951	0.940
2c	91.22	0.935	112.19	0.891	90.02	0.966	103.41	0.915	0.927
2d	97.54	1	125.94	1	93.16	1	113.03	1	1
2e	96.27	0.987	121.94	0.968	91.13	0.978	111.09	0.983	0.979
2f	90.43	0.927	115.8	0.919	87.99	0.945	102.96	0.911	0.926
2g	91.2	0.935	113.63	0.902	89.4	0.960	107.51	0.951	0.937
2h	91.15	0.934	112.91	0.897	83.75	0.899	108.75	0.962	0.923
2i	90.61	0.929	113.91	0.904	87.44	0.939	89.7	0.794	0.891
2j	90.43	0.927	114.88	0.912	85.31	0.916	103.24	0.913	0.917
2k	76.37	0.783	96.55	0.767	69.79	0.749	108.46	0.960	0.815

ASP, PLP (ChemPLP), CHS (Chemscore) e GS (Goldscore) - scoring functions; nASP, nPLP, nCHS e nGS, normalisation of scoring functions; nG - general normalisation of all functions.

**FIGURE 5:**
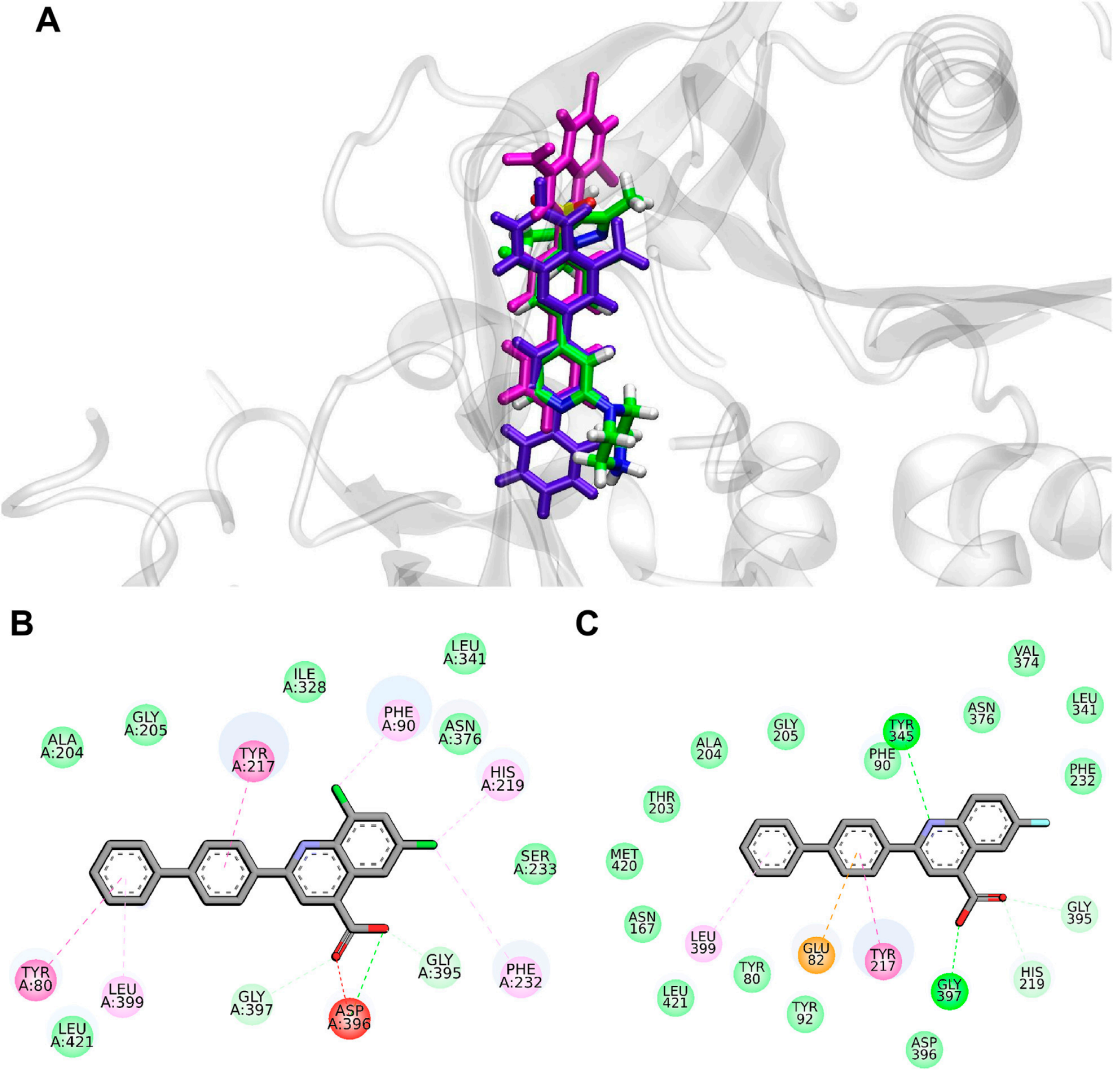
**(A)** Superimposition of the best docked pose of the crystallographic ligand, **DDD85646** (green), compound **1g** (magenta) and compound **2d** (purple) at *Lm*NMT binding site (PDB code 2WSA), and main interactions of the docked pose of the quinoline derivatives in *Ld*NMT: **(B) 1g**; **(C) 2d**. The color of interactions is represented by: π - π stacked in pink and π-alkyl in light pink; van der Waals interactions in light green; conventional hydrogen bonds in lime green; π-anion in orange; unfavorable interaction in red.

Figure 5A shows the comparison between the docking poses of **1g** and **2d** with the crystallographic ligand **DDD85646**. Compound **1g** was included in the analysis since it was used as the query molecule in the IVS approach.

Compound **2d** emerged as the most promising molecule of the dataset. Since there is *in vitro* antileishmanial activity data reported in the literature for *L. donovani* strain for similar molecules (Abdelwahid et al., 2019), docking simulations were performed to assess the binding affinity of compounds **2d** and **1g** for *Ld*NMT. The same pharmacophoric constraints from the 2WSA model were applied to *Ld*NMT (PDB code 2WUU) for docking, yielding a top Chemscore of 70.28 and 70.82, respectively. Discovery Studio 2021 analysis revealed interactions with key residues - Phe90, Tyr217, His219, and Asn376 - critical for *L. donovani* (Nascimento et al., 2023). Figures 5B,C represent the main interactions between the docked ligands **1g** and **2d**, respectively, and the binding site residues of *Ld*NMT.

### 3.5 Stability of NMT and quinoline derivatives complexes

**FIGURE 6:**
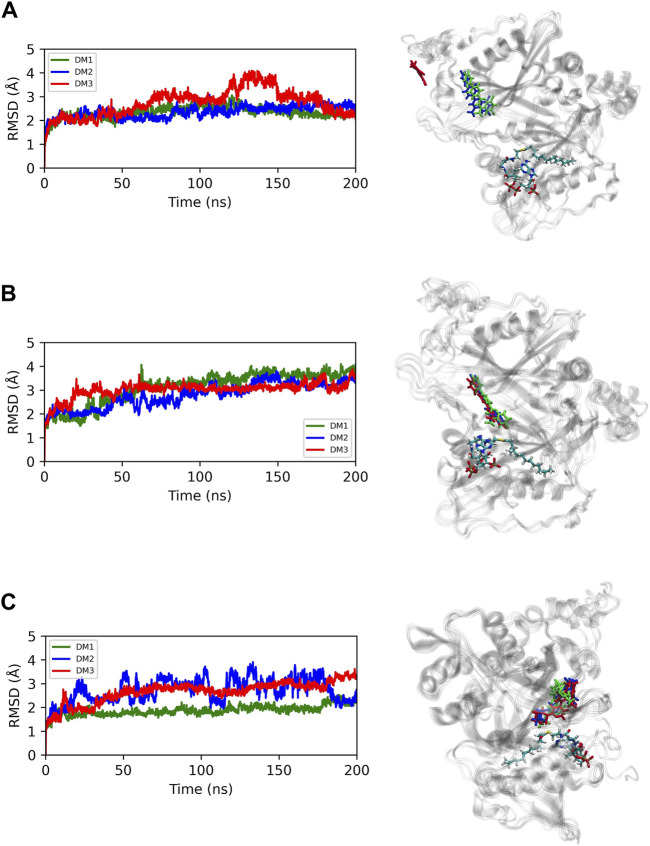
RMSDs plots of *Lm*NMT complexed with compounds and structures of the most representative complex: **(A)** 1g; **(B)** 2d and **(C)** DDD85646.

MD simulations were performed on *Lm*NMT to compare the dynamic behaviors of 1g and 2d to the crystallographic ligand (DDD85646). Using CPPTRAJ analysis tools, RMSD and RMSF plots were generated for the protein backbone atoms and each ligand individually. RMSD is a key parameter for assessing structural stability in MD simulations, analyzing both protein backbone and individual ligand movements. Protein backbone RMSD values remained below 4.0 Å, with compound 1g bound to *Lm*NMT showing a profile similar to the PDB ligand complex (Figure 6). Ligands bound to *Lm*NMT exhibited RMSD values under 2.0 Å, indicating a stable binding (left panel of Supplementary Figure S2A–C). Residue fluctuations (RMSF) were below 5 Å, with similar profiles across the three *Lm*NMT complexes (right panels of Supplementary Figure S2A–C). Peaks in residues ranging from 144 to 152 and from 235 to 245 correspond to non-active site regions. Initial peaks in the 1g complex relate to the enzyme’s surface region, while the active site residues showed fluctuations under 1.0 Å, demonstrating structural stability.

For clustering the MD trajectory, the first 100 ns of it were discarded due to the stability observed in the RMSD profiles of the *Lm*NMT complexes. Molecular docking was performed on the most representative protein conformation to assess ligand affinity, by using the same docking settings and the Chemscore function in GOLD. A significant increase in ligand affinity was observed for 1g in the most representative structure, suggesting that conformational changes induced by the structural flexibility during MD simulations enhanced ligand-receptor interactions. In contrast to compound 1g, compound 2d exhibited no significant improvement in binding affinity during the simulation. Ligand efficiency was calculated to assess binding affinity normalized by heavy atom count, reflecting the average contribution per atom rather than total compound affinity. Despite comparable binding affinities, compound 2d showed enhanced ligand efficiency over its analogues, suggesting more favorable atomic contributions to binding (Table 4).

**TABLE 4:** Comparison of the scores of the initial docked pose and the one at the most populated clusters, and ligand efficiency.

Compound	Initial score^a^	Score at the most populated cluster (cluster 1)^a^	Ligand efficiency
DDD85646	100.87	113.58	3.54
1g	72.53	95.04	3.52
2d	93.16	91.62	3.66

^a^ Docking scores obtained with Chemscore function in GOLD, software.

Using BIOVIA Discovery Studio, key interactions of the top docking poses in the most representative conformation were analyzed (Supplementary Figure S3). In both cases, π-π interactions with Tyr217, a residue critical for NMT’s mechanism in leishmaniasis, were observed. Additional crucial interactions for *L. major*, including van der Waals contacts with Tyr345, Thr203, and Gly205, were also identified (Nascimento et al., 2023).

### 3.6 ADMET properties

The pharmacokinetic and toxicity profiles of the series under study were evaluated and compared to the PDB ligand, DDD85646, which is a known inhibitor of *Lm*NMT. The results shown in Table 5 indicate that this new series presents promising pharmacokinetic characteristics and improved safety profiles in several key parameters. Solubility, a crucial factor influencing drug absorption and bioavailability, was significantly better in many of the quinolinic compounds. DDD85646 is poorly soluble, while compounds **1a**, **2a**, and **2h** to **2k**, were predicted to be soluble, and **1b**, **1d**ay, and **1e** as moderately soluble. Regarding lipophilicity, most compounds fall within the acceptable range (Log P ≤ 5), with only compound **1g** slightly exceeding this threshold, probably due to its biphenyl portion when compared to its analogues (see Supplementary Table S1).

**TABLE 5:** Pharmacokinetic and toxicity profiles (ADMET) of quinoline derivatives.

ID	Sol	Log P	GA	BBB	Gp-P	CYP3A4	AMES	Hepatotox
DDD85646	Poorly soluble	2.94	High	No	Yes	Yes	No	Yes
1a	Soluble	2.80	High	Yes	No	No	Yes	Yes
1b	Mod. Soluble	2.36	High	Yes	No	No	No	No
1c	Poorly soluble	3.34	High	Yes	No	No	No	No
1d	Mod. Soluble	2.37	High	Yes	No	No	No	No
1e	Mod. Soluble	2.99	High	Yes	No	No	No	No
1f	Poorly soluble	3.75	High	Yes	No	No	No	No
1g	Poorly soluble	5.14	High	No	Yes	No	Yes	Yes
2a	Soluble	3.12	High	No	No	No	No	Yes
2b	Poorly soluble	3.44	High	No	No	No	No	Yes
2c	Poorly soluble	3.44	High	No	No	No	No	Yes
2d	Poorly soluble	4.42	High	No	No	No	No	Yes
2e	Poorly soluble	4.05	High	No	No	No	No	Yes
2f	Poorly soluble	3.44	High	Yes	No	No	No	Yes
2g	Poorly soluble	3.74	High	No	No	No	No	Yes
2h	Soluble	2.67	High	No	No	No	No	Yes
2i	Soluble	2.71	High	No	No	No	No	Yes
2j	Soluble	3.09	High	No	No	No	No	Yes
2k	Soluble	2.97	High	No	No	No	Yes	Yes

Gastrointestinal absorption (GA) was predicted to be high for all compounds, indicating a generally favorable oral absorption profile across the series. BBB permeability predictions indicated that derivatives **1g**, **2a–2e**, and **2g–2k,** share the same profile as DDD85646 of not crossing BBB, which is a favorable feature for anti-leishmanial candidates against cutaneous and visceral forms of the disease, as it helps to minimize unnecessary CNS exposure and potential neurotoxicity. P-glycoprotein (gp-P) substrate status was positive only for DDD85646 and **1g**. All the other compounds are predicted not to be substrates, which is advantageous since gp-P can actively efflux drugs, reducing their intracellular concentrations and therapeutic effects. Notably, CYP3A4 inhibition, a concern for drug-drug interactions, was predicted only for DDD85646. The absence of predicted CYP3A4 inhibition in the remaining compounds reduces the risk of metabolic interactions and hepatotoxic effects due to impaired drug clearance.

Toxicity indicators such as AMES test and hepatotoxicity predictions provide complementary insights into the safety profile of this series. Regarding mutagenicity, DDD85646 was predicted to be non-mutagenic, a profile shared by most derivatives, including **1b** to **1f** and the majority of the 2-series. Only compounds **1a**, **1g** and **2k** showed positive AMES results, indicating potential mutagenic risk that requires further evaluation. In terms of hepatotoxicity, however, most compounds in the series were predicted to be hepatotoxic.

### 3.7 2-aryl-quinoline-4-carboxylic acid as a scaffold for new NMT inhibitors

The ligand-based similarity search in ChEMBL, ZINC and PubChem databases identified a set of structurally related compounds. Molecules were selected based on the following properties: soluble or moderately soluble, high gastrointestinal absorption (GA), and non-inhibition of the cytochrome P450 isoform (CYP3A4). Compounds identified as potential P-glycoprotein (P-gp) substrates were excluded due to the risk of cellular efflux issues. The retrieved molecules, shown in Supplementary Table S4, exhibit lower predicted binding affinity for NMT when compared to the compounds in the studied series, suggesting a superior affinity profile of the 2-aryl-quinoline-4-carboxylic acid derivatives series.

Additionally, the calculated ADMET properties for the retrieved compounds are summarized in Supplementary Table S5, supporting the drug-likeness and potential pharmacokinetic advantages of the 2-aryl-quinoline-4-carboxylic acid derivatives series. Although all molecules retrieved from the databases are predicted to be soluble, present low Log P values and high Gastrointestinal absorption (GA), all of them demonstrated potential to cross the blood-brain barrier. On the other hand, molecules predicted to have no interactions with gp-P and CYP3A4, and to be non-mutagenic and not hepatotoxic were found, except for mol357 which has an indicative of hepatotoxicity.

## 4 Discussion

Studies using the same IVS protocol confirmed, through enzyme inhibition assays, that the best target listed by IVS, in fact, presents the formation of the complex and intended inhibition (de Oliveira Viana et al., 2023; Lourenço et al., 2020). Therefore, the identification of NMT in our study can suggest it as the main target for inhibition in *Leishmania*. NMT is one of the few targets confirmed as genetically essential for the survival of *Leishmania* spp and that has also been pharmacologically validated as a drug target for *L. donovani* (Corpas-Lopez et al., 2018). NMT has been also evaluated in preclinical studies as a therapeutic target for the treatment of fungal and parasitic infections (Brannigan et al., 2014). In addition to having been characterized for the species *L. major* and *L. donovani*, this enzyme appears to be essential for the survival of promastigotes in both organisms (Brannigan et al., 2010; Price et al., 2003). Studies have shown that this inhibition leads to cellular dysfunction and parasite death (Rackham et al., 2015).

Although *in vitro* tests for similar compounds have been primarily performed in *L. donovani*, our computational predictions were carried out using the NMT from *L. major*. This choice was based on the availability of Ki data for *L. major* NMT ligand, which enables more robust docking protocol validation. Importantly, this enzyme shares over 90% sequence identity with *L. donovani* NMT, supporting its use as a structural model. Previous studies, such as Corpas-Lopez et al. (2018), have reported a modest correlation between enzyme inhibition and antiparasitic activity in axenic amastigotes, as well as limited selectivity over the human enzyme. Nevertheless, the results presented here offer a first step in identifying potential inhibitors targeting the conserved active site of *Leishmania* NMT and should be followed by biological validation in *L. donovani* and selectivity testing against the human enzyme.

Furthermore, previous studies have reported the importance of NMT in parasite survival, with this enzyme also present in *Trypanosoma cruzi*, *T. brucei*, *P. falciparum*, and *P. vivax* (Nascimento et al., 2023). Its reaction mechanism involves the lipid modification of proteins, directing them to membrane surfaces by catalyzing the transfer of a Myr-CoA (Myristoyl-CoA) to the N-terminus of glycine, resulting in the production of several cellular proteins. Thus, it is highlighted that NMT is essential for the viability of promastigotes and intracellular amastigotes of *L. donovani* and its interference has been a promising strategy in the design of drugs for human visceral leishmaniasis (Paape et al., 2020). As inhibitor development for NMT is focused on the pocket close to the Myr-CoA binding site, high-affinity inhibitors can be proposed to hinder interaction with the N-Gly terminus. Through this process, it is possible to prevent the N-myristoylation process and facilitate the design of selective inhibitors (Nascimento et al., 2023).

Studies by Kersten et al. (2020) indicate that the selectivity of potential inhibitors for *Lm*NMT over *Hs*NMT is linked to changes in residue flexibility rather than direct interactions with specific residues. The bulky active site of NMT allows the introduction of large groups in ligands to restrict flexibility in the C-terminal of *Hs*NMT, enhancing selectivity for *Lm*NMT. Compound **2d** is one of the bulkiest quinoline in the study, likely explaining its superior affinity for the *Lm*NMT active site compared to other molecules. Furthermore, the use of halogenated derivatives in aromatic moieties is supported by literature demonstrating their superior pharmacological properties compared to non-halogenated analogs, primarily due to enhanced compound stability (Musiol et al., 2006; Vamisetti et al., 2019). Halogens also form unique interactions with protein structures and side chains. Beyond this, their varying atomic sizes allow for precise molecular adjustments, optimizing inhibitors fit within specific enzymatic pockets (Vamisetti et al., 2019).

Certain NMT residues are crucial for the enzyme’s catalytic activity in leishmaniasis, including His219, Ser330, Tyr217 and Leu410. Additionally, in the catalytic mechanism, it is observed that the substrate binds to the site through interactions with Asn167 and Thr203 ​(Brannigan and Wilkinson, 2021). Thus, compounds containing the quinoline core may act as competitive inhibitors of NMT, blocking its function and preventing the myristoylation of essential proteins. The quinoline structure allows hydrophobic interactions and π-π stacking with residues of the NMT enzyme, increasing its affinity for the active site (Goncalves et al., 2017). In our study, these residues were shown to interact with our quinoline derivatives, emphasizing the compound’s importance in its inhibitory activity.

Similar results were found in the literature. Romero et al. (2019) evaluated a series of 4-aminoquinolines against *Leishmania braziliensis* and *Leishmania mexicana*, with compounds showing IC_50_ values from 3.84 to 10 μM. In the mechanism of action and molecular simulated studies, the NMT enzyme was identified as having the best interaction with the compounds, similar to the interactions in our study. In a separate study, Huang et al. (2021) conducted virtual screening of selenide compounds for antileishmanial activity, finding that quinoline derivatives had the strongest interaction with NMT, suggesting its inhibition as potential mechanism.

Previous studies have highlighted that, despite showing potent *in vitro* inhibition, NMT inhibitors have rarely translated into effective *in vivo* antileishmanial therapies. As discussed by Marín et al. (2024) and Brannigan and Wilkinson (2021), these failures are often attributed to pharmacokinetic limitations, poor subcellular distribution, and insufficient accumulation of compounds within infected macrophages, where the parasite resides. In this context, our study aimed to explore whether the proposed quinoline derivatives could offer improved predicted binding affinity and drug-like properties, which are essential features for overcoming some of these limitations. While our computational predictions do not account for species-specific differences in NMT biology or tissue localization, we recognize that the contrasting clinical profiles of *L. major* (cutaneous) and *L. donovani* (visceral) may influence the *in vivo* efficacy of NMT inhibition. These aspects underscore the importance of integrating phenotypic assays in future work to validate the potential of NMT-targeted compounds across different species and disease forms.

The pharmacokinetic and toxicity profiles of the 2-aryl-quinoline-4-carboxylic acid derivatives reveal important improvements over DDD85646. Enhanced solubility, high predicted gastrointestinal absorption, and the absence of CYP3A4 inhibition and P-glycoprotein substrate status in most compounds support their potential for oral use and reduced risk of drug interactions. The lack of blood-brain barrier permeability is favorable for treating leishmaniasis. In parallel, a ligand-based similarity search in ChEMBL, ZINC, and PubChem databases identified structurally related compounds with good predicted solubility, low Log P, and high gastrointestinal absorption, but all exhibited potential to cross the blood-brain barrier. Although hepatotoxicity predictions remain a concern, the overall profiles suggest that 2-aryl-quinoline-4-carboxylic acid derivatives represent promising candidates for further development.

## 5 Conclusion

This study provides robust computational evidence supporting *L. major* N-myristoyltransferase (*Lm*NMT) as the primary molecular target of a novel series of 2-aryl-quinoline-4-carboxylic acid derivatives. The integration of inverse virtual screening, molecular docking, molecular dynamics, and ligand-based similarity analysis revealed consistent binding stability and favorable interactions with *Lm*NMT. Compound **2d** emerged as a particularly promising candidate, exhibiting strong binding affinity and ligand efficiency. In parallel, compound **1g** demonstrated enhanced interaction potential following conformational adaptation of the enzyme. Compared to structurally related compounds retrieved from public databases, the studied derivatives displayed superior predicted affinity for *Lm*NMT. Furthermore, the pharmacokinetic and toxicity predictions highlight drug-like properties across the series, suggesting good oral bioavailability and manageable safety risks. Despite the known challenges associated with NMT inhibitors in *Leishmania*, the compounds proposed in this study contribute to the expansion of chemical space around this validated target, reinforcing the role of target-based approaches as a starting point for drug discovery when combined with medicinal chemistry strategies and phenotypic assays. These findings not only reinforce the quinoline scaffold as a promising basis for antileishmanial drug development but also lay the groundwork for future experimental validation and lead optimization targeting *Lm*NMT.

## Data Availability

The original contributions presented in the study are included in the article/Supplementary Material, further inquiries can be directed to the corresponding authors.
