# Supplementary material for: In silico target identification and pharmacokinetic profiling of 2-aryl-quinoline-4-carboxylic acid derivatives as potential antileishmanial agents
Source: Front Pharmacol. 2025 Jul 21;16:1621059. doi: 10.3389/fphar.2025.1621059 (PMC12319028; doi:10.3389/fphar.2025.1621059)
Supplement: Supplementary file 1 [file DataSheet1.docx]

Supplementary Material

In silico target identification and pharmacokinetic profiling of 2-aryl-quinoline-4-carboxylic acid derivatives as potential antileishmanial agents

Marília Cecília da Silva^1^, Jéssika de Oliveira Viana^1*^, Tayná Rodrigues Olegário^2^, Jayne Maria Sabino^2^, Euzébio Guimarães Barbosa^3^, Elton José Ferreira Chaves^1^, Gerd Bruno Rocha^1^, Claudio Gabriel Lima-Junior^2^*, Karen Cacilda Weber^1^*

# Supplementary Tables

**Table S1.** Structures of 2-aryl-quinoline-4-carboxylic acid derivatives employed as a dataset (Muscia et al., 2009; Abdelwahid et al., 2019; and Olegário et al., 2025).

| **ID** | **Structure** | **Smile code** |
| --- | --- | --- |
| 1a | 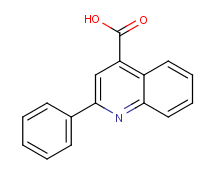 | OC(=O)c1cc(nc2c1cccc2)c1ccccc1 |
| 1b | 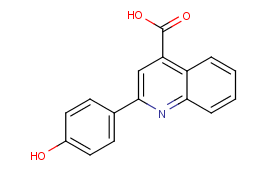 | Oc1ccc(cc1)c1nc2ccccc2c(c1)C(=O)O |
| 1c | 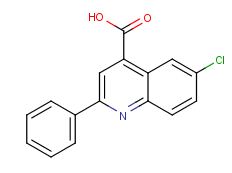 | Clc1ccc2c(c1)c(cc(n2)c1ccccc1)C(=O)O |
| 1d | 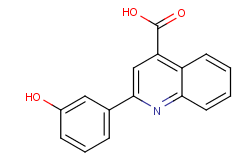 | Oc1cccc(c1)c1nc2ccccc2c(c1)C(=O)O |
| 1e | 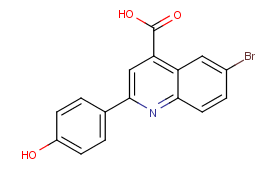 | Oc1ccc(cc1)c1nc2ccc(cc2c(c1)C(=O)O)Br |
| 1g | 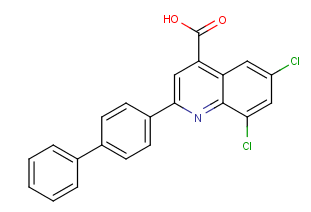 | Clc1cc(Cl)c2c(c1)c(cc(n2)c1ccc(cc1)c1ccccc1)C(=O)O |
| 1f | 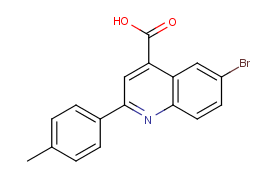 | Cc1ccc(cc1)c1nc2ccc(cc2c(c1)C(=O)O)Br |
| 2a | 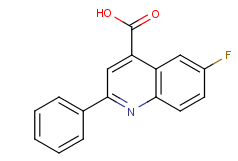 | Fc1ccc2c(c1)c(cc(n2)c1ccccc1)C(=O)O |
| 2b | 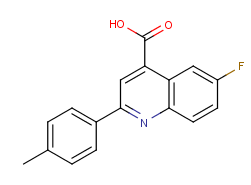 | Cc1ccc(cc1)c1nc2ccc(cc2c(c1)C(=O)O)F |
| 2c | 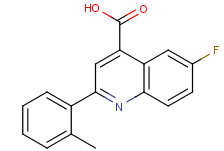 | Fc1ccc2c(c1)c(cc(n2)c1ccccc1C)C(=O)O |
| 2d | 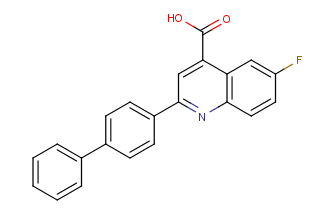 | Fc1ccc2c(c1)c(cc(n2)c1ccc(cc1)c1ccccc1)C(=O)O |
| 2e | 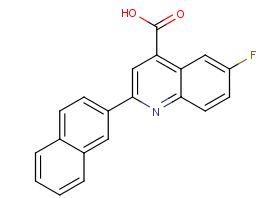 | Fc1ccc2c(c1)c(cc(n2)c1ccc2c(c1)cccc2)C(=O)O |
| 2f | 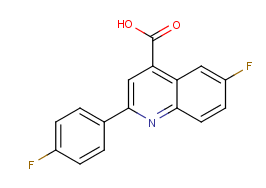 | Fc1ccc(cc1)c1nc2ccc(cc2c(c1)C(=O)O)F |
| 2g | 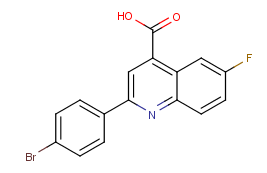 | Brc1ccc(cc1)c1nc2ccc(cc2c(c1)C(=O)O)F |
| 2h | 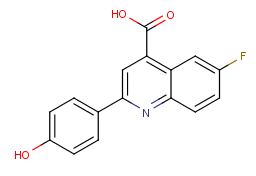 | Oc1ccc(cc1)c1nc2ccc(cc2c(c1)C(=O)O)F |
| 2i | 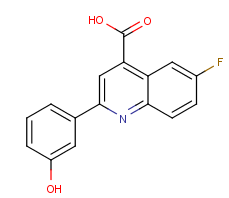 | Fc1ccc2c(c1)c(cc(n2)c1cccc(c1)O)C(=O)O |
| 2j | 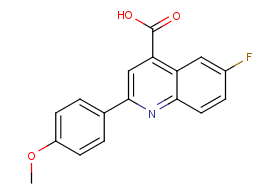 | COc1ccc(cc1)c1nc2ccc(cc2c(c1)C(=O)O)F |
| 2k | 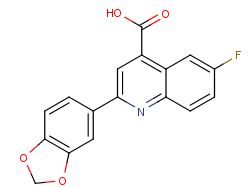 | Fc1ccc2c(c1)c(cc(n2)c1ccc2c(c1)OCO2)C(=O)O |

**Table S2.** Molecules retrieved from the ChEMBL database.

| **ChEMBL ID** | **K_i_ (nM)** | **ChEMBL ID** | **K_i_ (nM)** |
| --- | --- | --- | --- |
| CHEMBL4532268 | 1.3 | CHEMBL3912318 | 19.8 |
| CHEMBL3357688 | 1.4 | CHEMBL3770219 | 24.8 |
| CHEMBL3912318 | 1.6 | CHEMBL4532268 | 36.6 |
| CHEMBL4532268 | 2.0 | CHEMBL1230468 | 41.3 |
| CHEMBL3912318 | 2.3 | CHEMBL4435880 | 52.3 |
| CHEMBL3357688 | 6.0 | CHEMBL3754110 | 70.8 |
| CHEMBL4470652 | 7.3 | CHEMBL3344387 | 280 |
| CHEMBL1230468 | 8.4 | CHEMBL3344386 | 520 |
| CHEMBL3357688 | 8.5 | CHEMBL3344386 | 980 |
| CHEMBL1230468 | 13.9 | CHEMBL3344386 | 2600 |
| CHEMBL4449105 | 19.6 | - | - |

**Table S3.** Best ranked targets identified by IVS.

| **PDB ID** | **Score** | **Protein** | **Ligand** | **Species** |
| --- | --- | --- | --- | --- |
| 5A28 | -12.3 | N-myristoyltransferase | TUQ | *L. major* |
| 4CGN | -12.3 | N-myristoyltransferase | 7AH | *L. major* |
| 4CYQ | -11.6 | N-myristoyltransferase | YAU | *L. major* |
| 4CYN | -11.6 | N-myristoyltransferase | A6M | *L. major* |
| 4CYO | -11.4 | N-myristoyltransferase | UEK | *L. major* |
| 4CGO | -11.4 | N-myristoyltransferase | 6KV | *L. major* |
| 4CYP | -11.2 | N-myristoyltransferase | A62 | *L. major* |
| 4CGM | -11.1 | N-myristoyltransferase | CWZ | *L. major* |
| 6EWF | -11.0 | N-myristoyltransferase | 31A | *L. major* |
| 5G20 | -10.7 | N-myristoyltransferase | QBY | *L. major* |
| 5G21 | -10.6 | N-myristoyltransferase | YN4 | *L. major* |
| 5A27 | -10.6 | N-myristoyltransferase | TUT | *L. major* |
| 1W0C | -10.5 | Pteridine reductase | TAQ | *L. major* |
| 5USF | -10.4 | Tyrosyl-tRNA synthetase | YSA | *L. donovani* |
| 6GNV | -10.1 | N-myristoyltransferase | F5E | *L. major* |
| 6GNU | -10.1 | N-myristoyltransferase | F5Z | *L. major* |
| 6GNT | -10.1 | N-myristoyltransferase | F5Q | *L. major* |
| 6GNS | -10.1 | N-myristoyltransferase | F65 | *L. major* |
| 6GNH | -10.1 | N-myristoyltransferase | F4T | *L. major* |
| 6EU5 | -9.70 | N-myristoyltransferase | BXN | *L. major* |
| 5AG6 | -9.70 | N-myristoyltransferase | 5PE | *L. major* |
| 5AGE | -9.60 | N-myristoyltransferase | M9M | *L. major* |
| 4A33 | -9.40 | N-myristoyltransferase | PS8 | *L. major* |
| 2WSA | -9.30 | N-myristoyltransferase | 646 | *L. major* |

**Table S4.** Structural representations of the highest-scoring compounds identified through database screening.

| **ID** | **Database** | **Structure** | **Score** |
| --- | --- | --- | --- |
| mol357 | Pubchem | 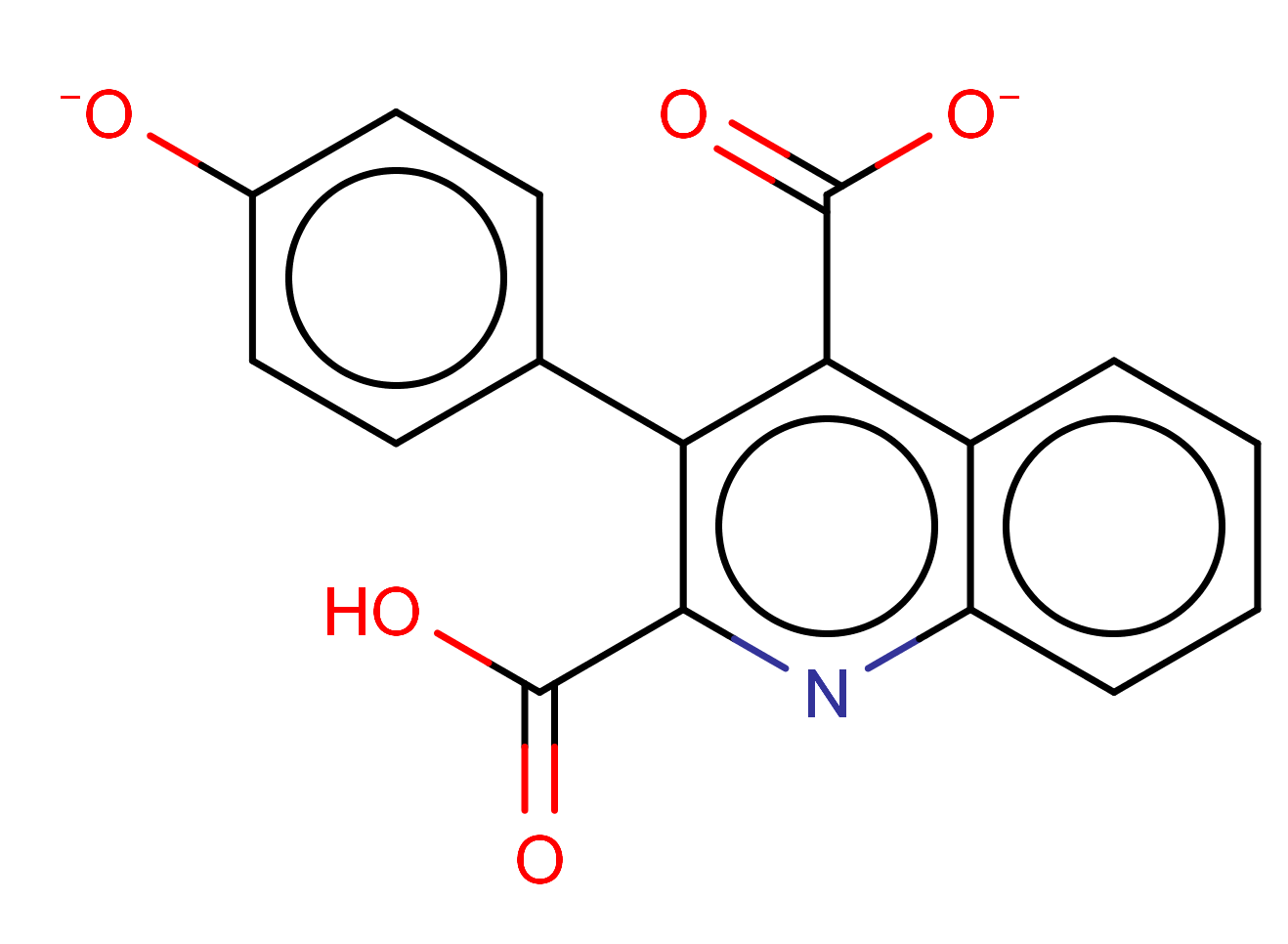 | 65.48 |
| mol15 | Pubchem | 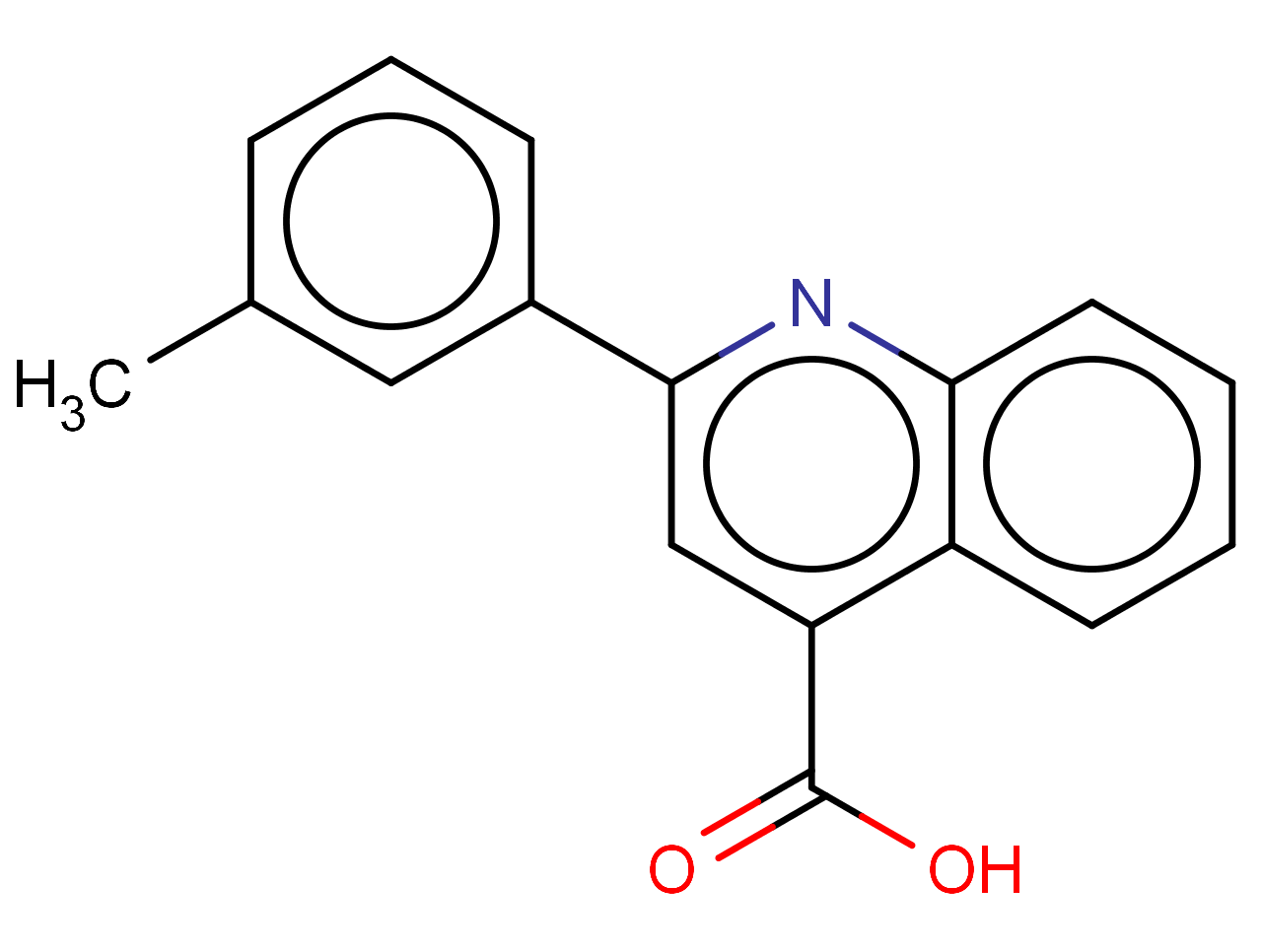 | 65.10 |
| mol197 | Pubchem | 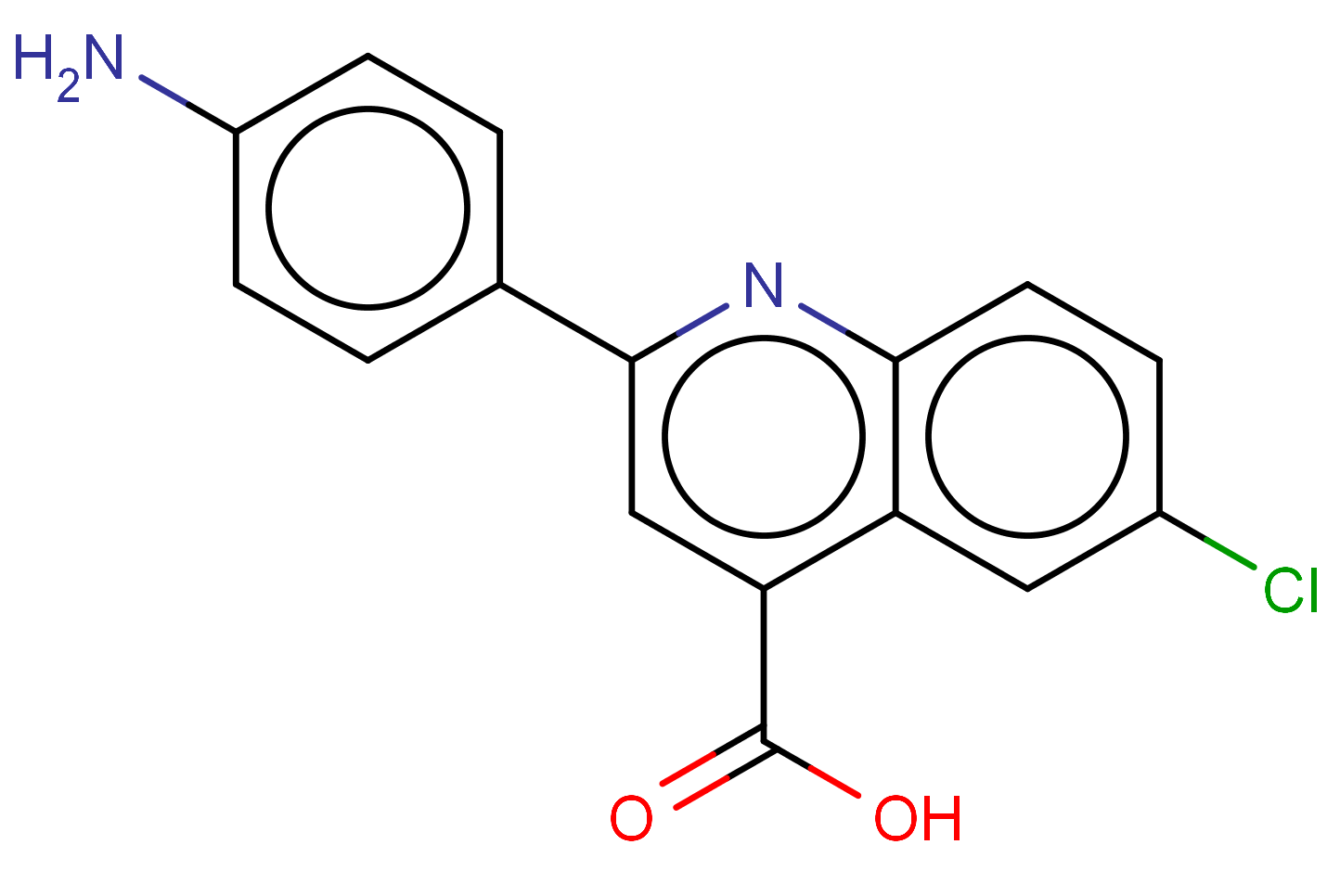 | 61.34 |
| mol198 | Pubchem | 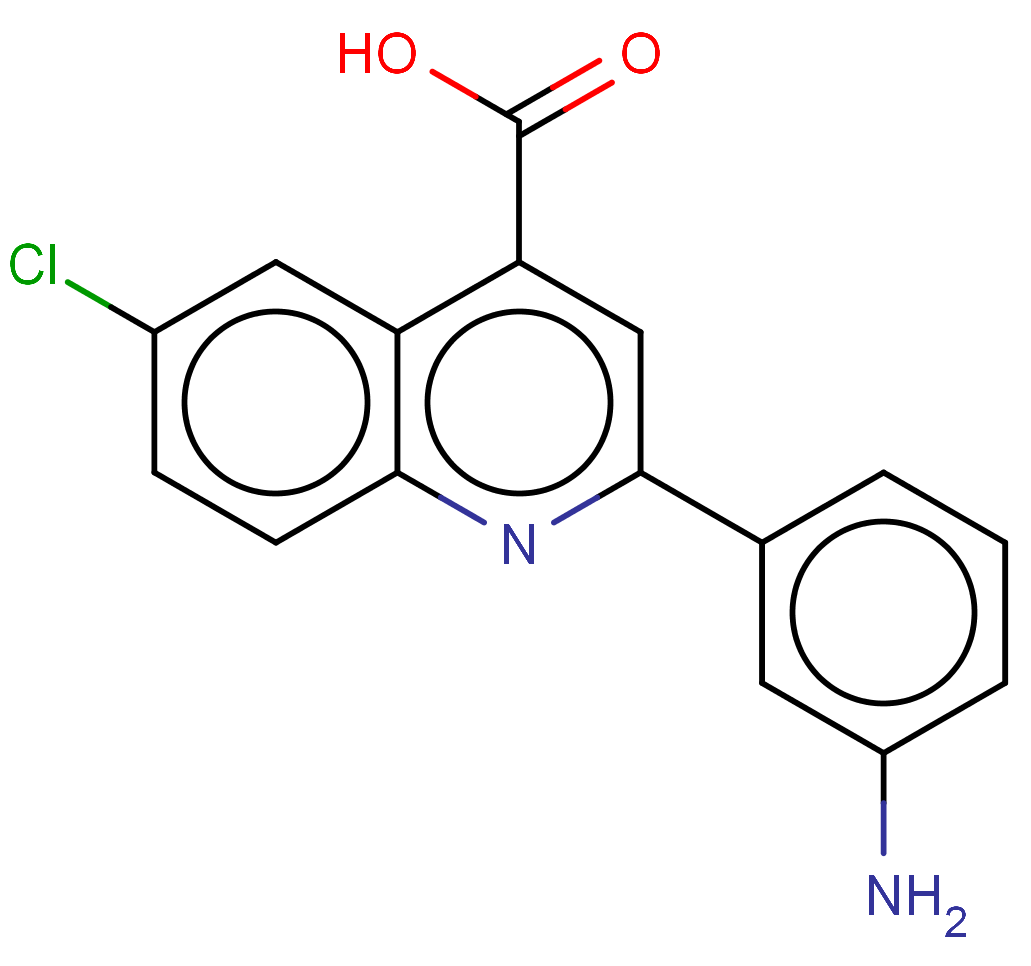 | 60.46 |
| CHEMBL1992779 | ChEMBL | 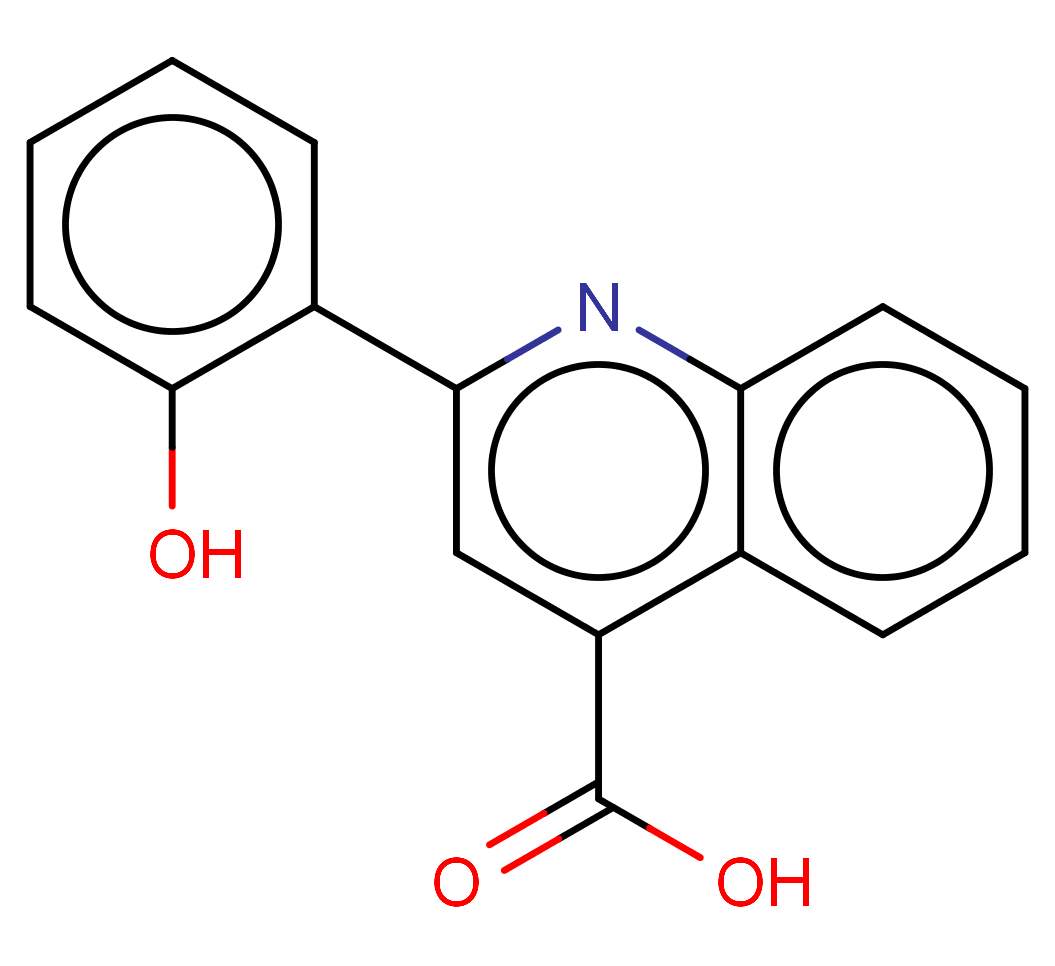 | 60.09 |
| CHEMBL2172252 | ChEMBL | 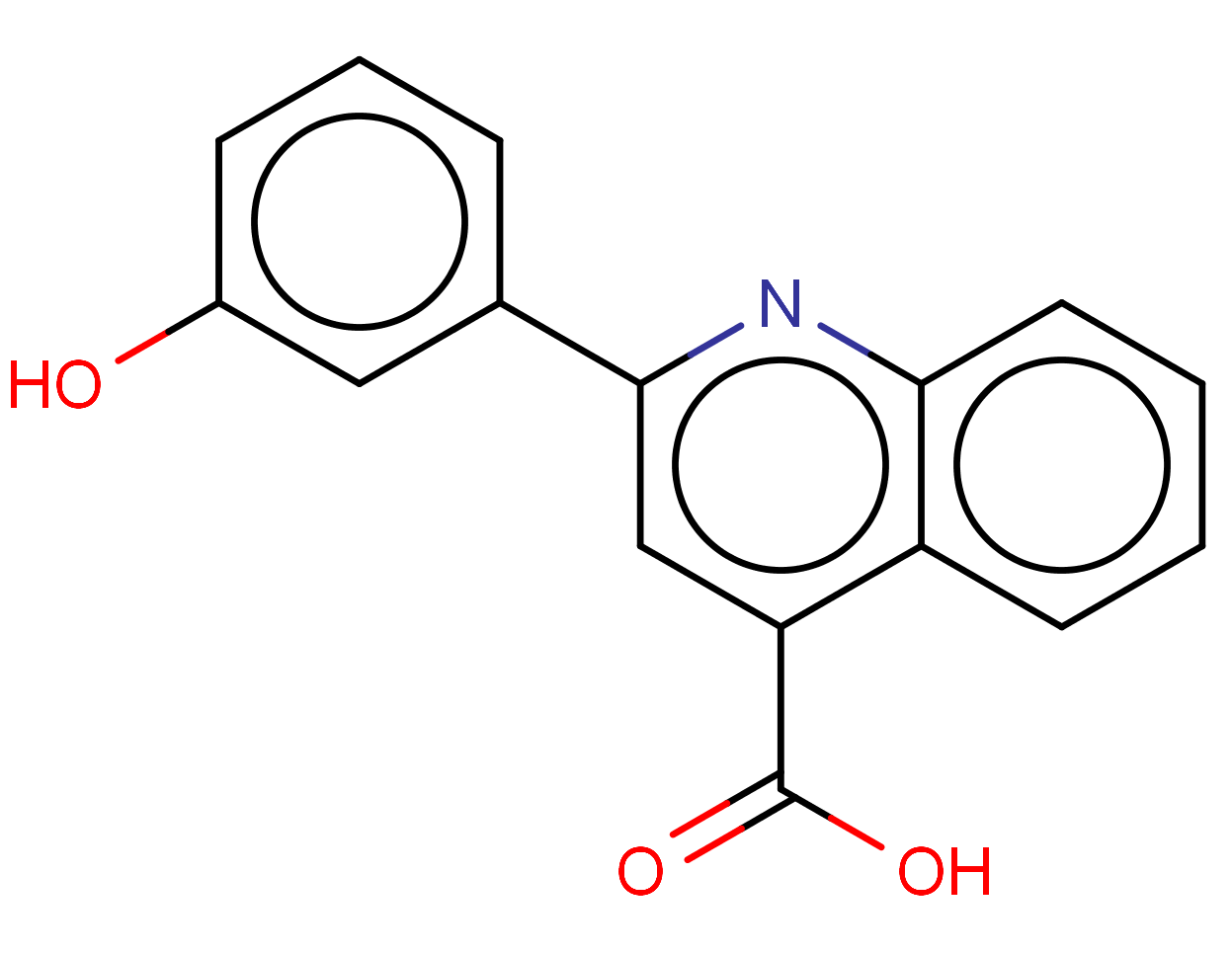 | 59.48 |
| CHEMBL1408157 | ChEMBL | 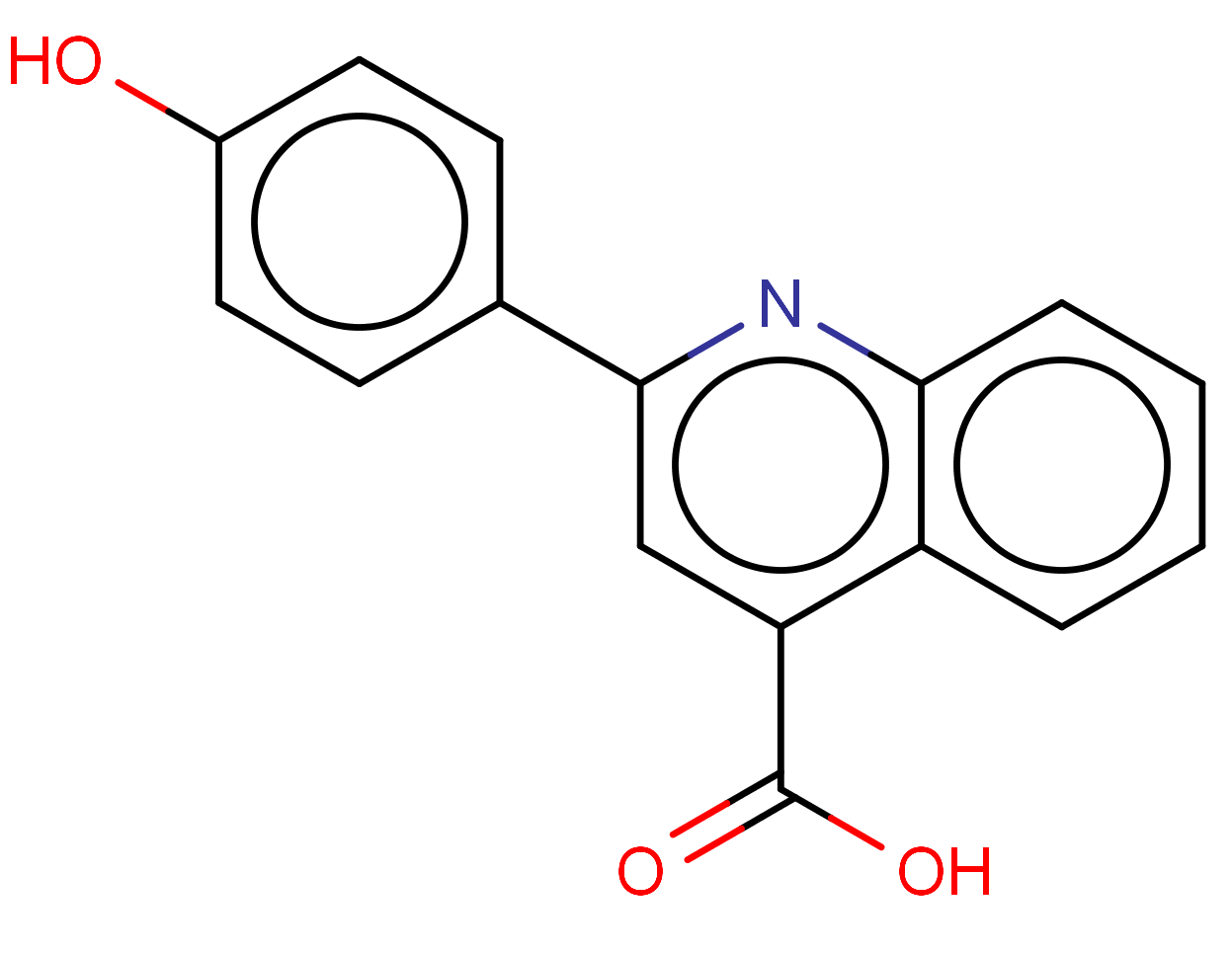 | 59.33 |

**Table S5.** Pharmacokinetic and toxicity profiles (ADMET) of the compounds identified through screening.

| **ID** | Sol. | Log P | GA | BBB | gp-P | CYP3A4 | AMES | Hepatotox. |
| --- | --- | --- | --- | --- | --- | --- | --- | --- |
| mol357 | Soluble | 1.97 | High | Yes | No | No | No | Yes |
| mol15 | Soluble | 3.12 | High | Yes | No | No | No | No |
| mol197 | Soluble | 2.80 | High | Yes | No | No | No | No |
| mol198 | Soluble | 2.77 | High | Yes | No | No | No | No |
| CHEMBL2172252 | Soluble | 2.37 | High | Yes | No | No | No | No |
| CHEMBL1408157 | Soluble | 2.36 | High | Yes | No | No | No | No |
| CHEMBL1992779 | Soluble | 2.32 | High | Yes | No | No | No | No |

# Supplementary Figures

**A**


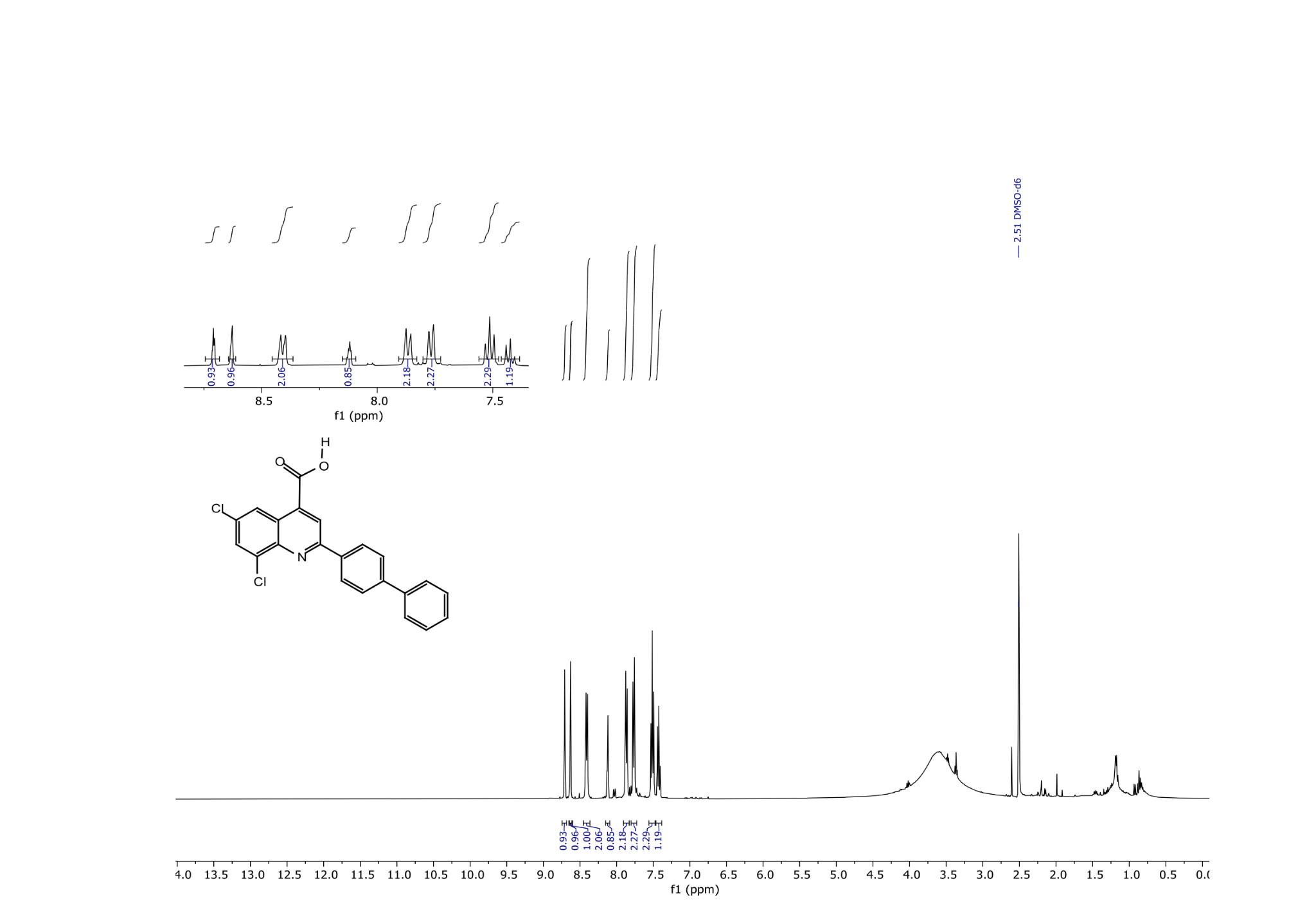


**B**


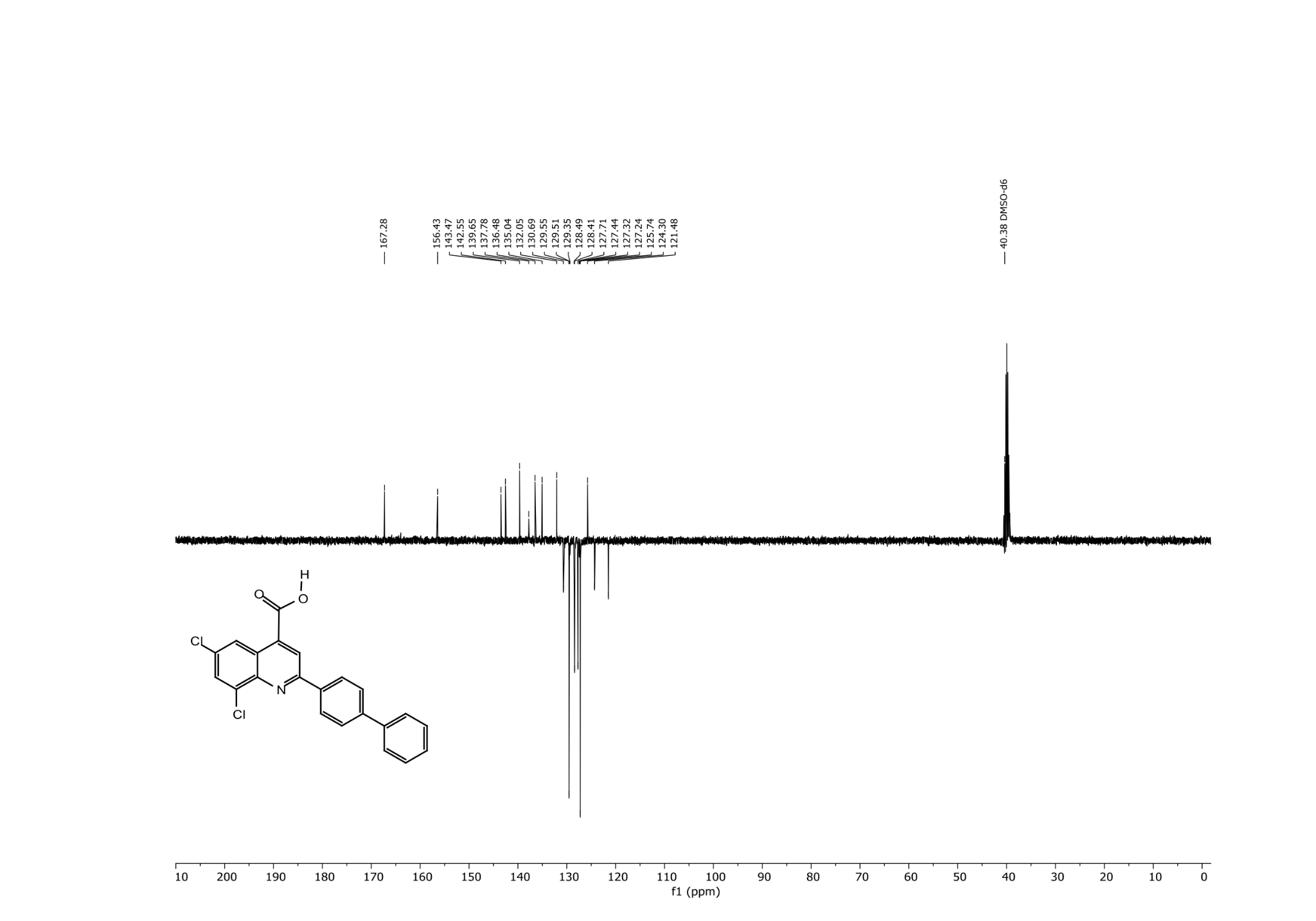


**Figure S1**. **(A)** ^1^H NMR spectra (400 MHz, DMSO-*d6*) of compound **1g**; (B) ^13^C-APT NMR spectra (100 MHz, DMSO-*d6*) of compound **1g**.


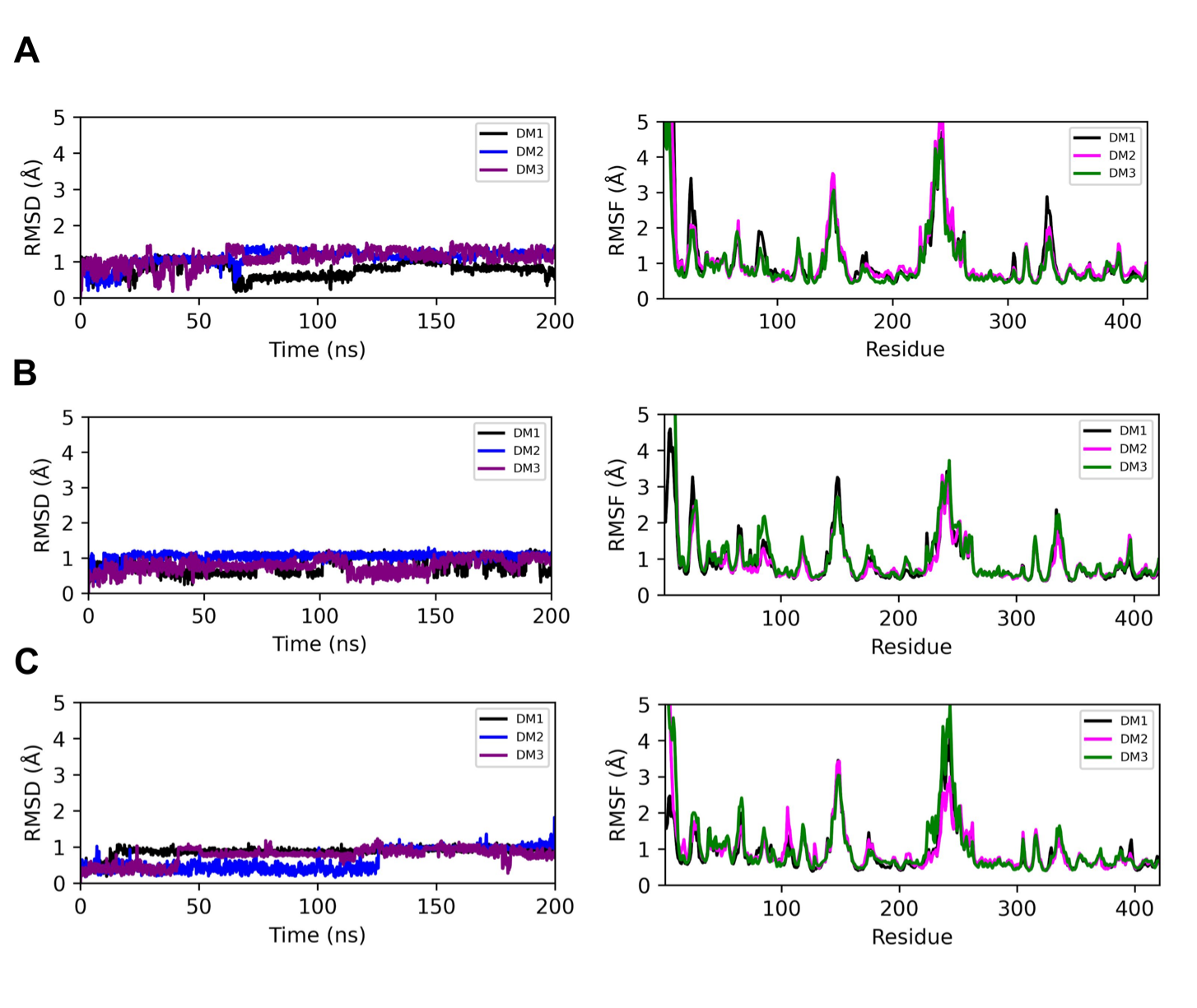


**Figure S2.** RMSD (left) and RMSF (right) plots for the three MD replicas of the compounds complexed with *Lm*NMT. (**A)** 1g, (**B)** 2d and (**C)** DDD85646.

| **A**  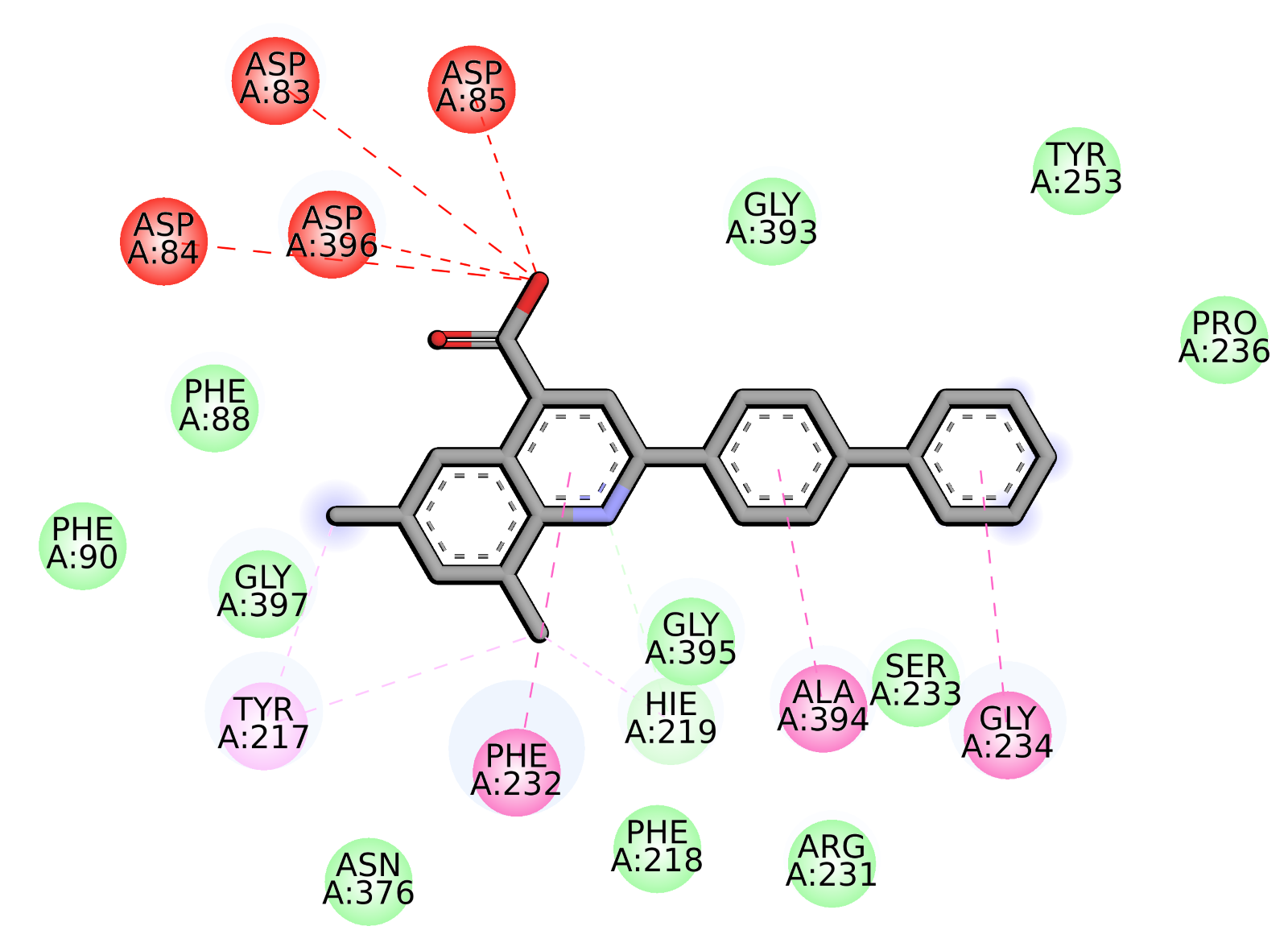 | **B**  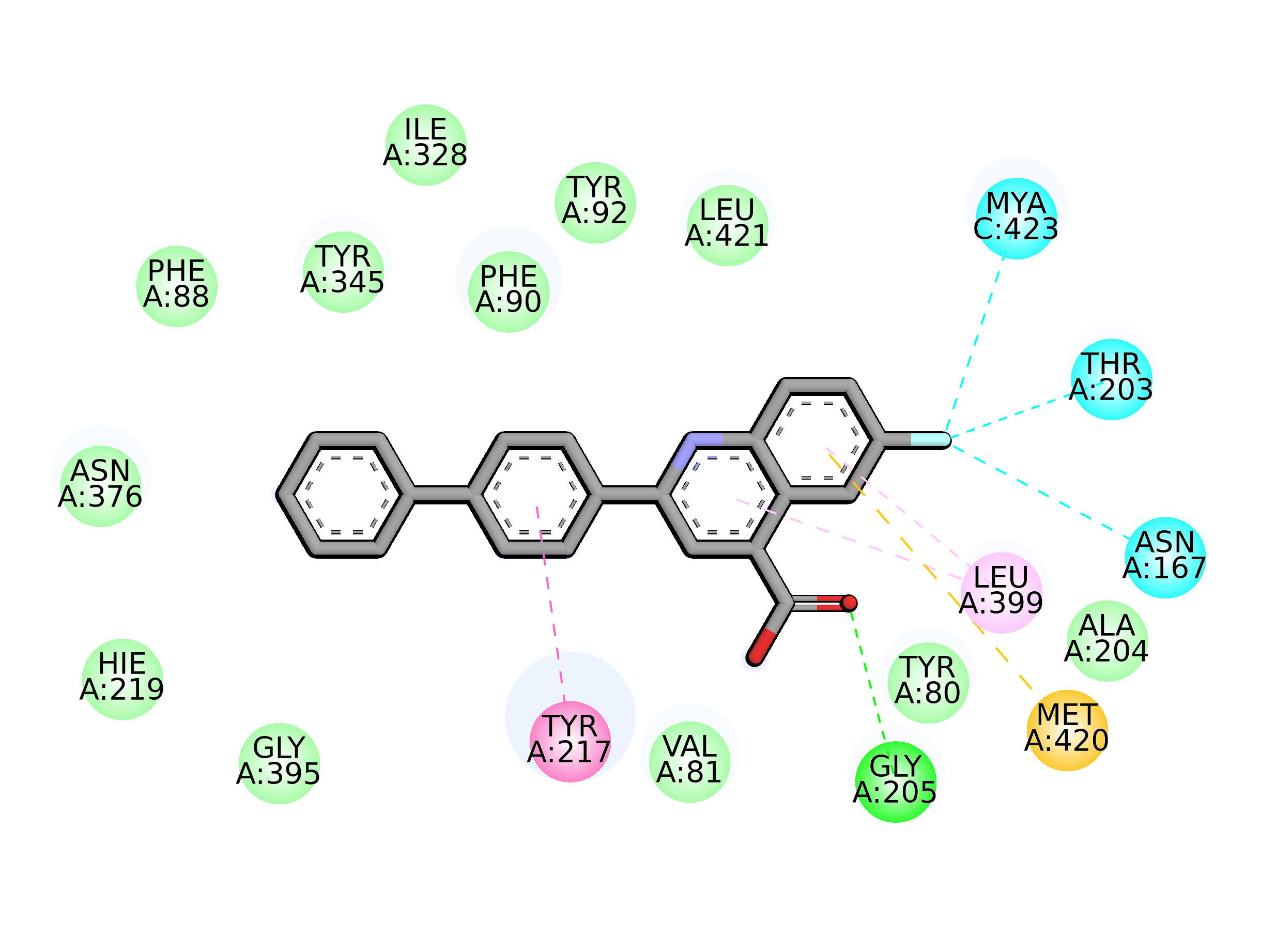 |
| --- | --- |

**Figure S3.** Main interactions of the docked poses of quinolines compounds in the most representative conformation of *Lm*NMT enzyme: (**A**) 1g, (**B)** (2d). The color of interactions is represented by: π - π stacked in pink and π-alkyl in light pink; van der Waals interactions in light green; conventional hydrogen bonds in lime green; π-anion in orange; unfavorable interaction in red.
